# A cure for the blues: opsin duplication and subfunctionalization for short-wavelength sensitivity in jewel beetles (Coleoptera: Buprestidae)

**DOI:** 10.1186/s12862-016-0674-4

**Published:** 2016-05-18

**Authors:** Nathan P. Lord, Rebecca L. Plimpton, Camilla R. Sharkey, Anton Suvorov, Jonathan P. Lelito, Barry M. Willardson, Seth M. Bybee

**Affiliations:** Department of Biology, Brigham Young University, 4102 LSB, Provo, UT 84602 USA; Department of Chemistry and Biochemistry, Brigham Young University, C100 BNSN, Provo, UT 84602 USA; United States Department of Agriculture, Animal and Plant Health Inspection Service, Plant Protection and Quarantine, Emerald Ash Borer Program, 5936 Ford Court Suite 200, Brighton, MI 48116 USA

**Keywords:** Insect vision, Opsins, *Agrilus planipennis*, Emerald ash borer, Pest species, RNA-seq, Transcriptome, Evolution

## Abstract

**Background:**

Arthropods have received much attention as a model for studying opsin evolution in invertebrates. Yet, relatively few studies have investigated the diversity of opsin proteins that underlie spectral sensitivity of the visual pigments within the diverse beetles (Insecta: Coleoptera). Previous work has demonstrated that beetles appear to lack the short-wavelength-sensitive (SWS) opsin class that typically confers sensitivity to the “blue” region of the light spectrum. However, this is contrary to established physiological data in a number of Coleoptera. To explore potential adaptations at the molecular level that may compensate for the loss of the SWS opsin, we carried out an exploration of the opsin proteins within a group of beetles (Buprestidae) where short-wave sensitivity has been demonstrated. RNA-seq data were generated to identify opsin proteins from nine taxa comprising six buprestid species (including three male/female pairs) across four subfamilies. Structural analyses of recovered opsins were conducted and compared to opsin sequences in other insects across the main opsin classes—ultraviolet, short-wavelength, and long-wavelength.

**Results:**

All nine buprestids were found to express two opsin copies in each of the ultraviolet and long-wavelength classes, contrary to the single copies recovered in all other molecular studies of adult beetle opsin expression. No SWS opsin class was recovered. Furthermore, the male *Agrilus planipennis* (emerald ash borer—EAB) expressed a third LWS opsin at low levels that is presumed to be a larval copy. Subsequent homology and structural analyses identified multiple amino acid substitutions in the UVS and LWS copies that could confer short-wavelength sensitivity.

**Conclusions:**

This work is the first to compare expressed opsin genes against known electrophysiological data that demonstrate multiple peak sensitivities in Coleoptera. We report the first instance of opsin duplication in adult beetles, which occurs in both the UVS and LWS opsin classes. Through structural comparisons of known insect opsins, we suggest that opsin duplication and amino acid variation within the chromophore binding pocket explains sensitivity in the short-wavelength portion of the visible light spectrum in these species. These findings are the first to reveal molecular complexity of the color vision system within beetles.

**Electronic supplementary material:**

The online version of this article (doi:10.1186/s12862-016-0674-4) contains supplementary material, which is available to authorized users.

## Background

Vision is central to many important biological and behavioral processes such as navigation, mate selection, predator avoidance, and foraging. The sensitivity of visual systems to different regions of the visible light spectrum is achieved through the interaction of light photons with visual pigment molecules housed in the photoreceptor cells of the eye. Each visual pigment molecule is composed of a G-protein coupled receptor—an opsin—bound to a photosensitive chromophore, retinal. Previous studies have suggested substitutions of amino acids in the seven trans-membrane domains closest to the chromophore—the chromophore binding pocket—have the highest potential for altering the peak spectral sensitivity (λ_max_ value) of a visual pigment, known as spectral tuning (see [1–11]). There are two key aspects to the opsin chromophore binding pocket that determine the spectral sensitivity of retinal isomerization upon light activation. The first is the chemical environment of the protonated Schiff base, and the second is the shape of the chromophore, which is determined by the structure of the chromophore binding pocket. Amino acid substitutions in this region may alter the geometry and steric space available for binding the chromophore and/or alter the chemical nature and hydrogen bonding in the pocket [12–15]. Thus, the identification of amino acid variation between putative opsin copies is an important step in predicting spectral tuning of photopigments.

Insect opsins, which underpin sensitivity to ultraviolet (~350 nm), short (~440 nm), and long (~530 nm) wavelengths [6], form natural phylogenetic groups called opsin classes. The common ancestor of the insects is hypothesized to have been trichromatic and possessed a single opsin of each spectral class (ultraviolet sensitive UVS, short-wavelength sensitive SWS, and long-wavelength sensitive LWS) [6, 16, 17]. Duplications of the LWS and/or SWS opsin classes have been shown in a number of insect orders (e.g., Odonata: [18]; Orthoptera: [19]; Lepidoptera: [20–23]; Diptera: [24–26]; Hymenoptera: [27]). However, UVS duplications among insects are much less common and have only been recorded within a few members of Lepidoptera (*Heliconius*, e.g., [28]), Diptera (e.g., [29]), Hemiptera (e.g., [30–32]), and Coleoptera (first larval instar only in *Thermonectus marmoratus* Gray, [33]). These duplications have also been shown to result in greater ability to discriminate ultraviolet signals (e.g., [34]). Conspicuously absent, however, are detailed studies on the most diverse animal order—Coleoptera (beetles).

### Coleoptera visual systems—a loss of short-wavelength sensitivity?

Opsin sequence data for Coleoptera is sparse, having only been generated for three groups to date (Tenebrionidae: *Tribolium castaneum* (Herbst) [35–38]; Dytiscidae [33, 39, 40]; Lampyridae [41–43]). Interestingly, these studies only recovered two opsin classes (UVS and LWS) and failed to recover a SWS opsin, suggesting the loss of an entire opsin class. Such a loss is rare, having only been reported in four other species of insects [6, 44–46].

Most physiological studies on coleopteran eyes support the loss of a SWS opsin class, only detecting sensitivities in the UV and long wavelengths (e.g., [39, 47–57]; Table 1). A few studies, however, have demonstrated the presence of three to five peak spectral sensitivities in other beetle groups (Buprestidae: [58]; Cantharidae [59]; Carabidae: [60]; Chrysomelidae: [61]; Coccinellidae [62]; Glaphyridae: [63]; Lampyridae: [41]; Rhagophthalmidae [64]; Scarabaeidae: [65]). Such diversity in spectral sensitivity suggests that the visual systems of these species have a greater underlying molecular complexity. Other spectral tuning mechanisms, such as filtering pigments in fireflies [52, 66], serve to narrow and shift the spectral sensitivity of the visual pigment by small amounts (~20–25 nm). However, to maintain high photon catch, large (~100 nm) shifts in peak sensitivity would be better served by a dedicated photopigment. Thus, the aim of this paper is to explore the potential molecular diversity in beetles that might explain the diverse spectral sensitivities observed. As yet, complementary studies with both physiological and molecular data only exist for fireflies (Lampyridae) [41–43] and diving beetles (Dytiscidae) [33, 39, 40], both of which lack sensitivity to short wavelengths.

**Table 1:** Summary of spectral sensitivities (λmax) from previous studies on Coleoptera

Family	Taxon	Spectral sensitivities (in nm)	Method	Reference
Buprestidae	*Agrilus planipennis*	340, 420–430, 460, 540–560, 640–670	ERG	58
Cantharidae	*Chauliognathus pulchellus*	360, 450, 520–530	IntCell	59
Carabidae	*Carabus nemoralis*	348, 430, 500, 620	ERG	60
	*Carabus auratus*	348, 430, 500, 620	ERG	60
	*Cicindela chinensis*	525	ERG	56
	*Cincindela specularis*	360–380, 510–530	ERG	57
	*Cincindela japonica*	360–380, 510–530	ERG	57
Chrysomelidae	*Leptinotarsa decimlineata*	370, 450, 530	ERG & IntCell	61
Coccinellidae	*Coccinella septumpunctata*	360, 420, 520	IntCell	62
Curculionidae	*Dendroctonus pseudotsugae*	450, 510–530	ERG	50
	*Ips paraconfusus*	450, 510–530	ERG	50
Dytiscidae	*Thermonectus marmoratus*	375, 520	IntCell	39
Elateridae	*Pyrophorus punctatissimus*	near UV, 545	ERG	54
Glaphyridae	*Pygopleurus israelitus*	360, 517, 631	ERG	63
	*Photuris lucicrescens*	350, 440, 550	ERG	41
Lampyridae	*Various genera and species*	360–420, 550–580	ERG	48, 51-53, 55
Rhagophthalmidae	*Rhagophthalmus ohbai*	360, 540–560, 600	ERG	64
Scarabaeidae	*Anomala corpulenta*	400, 460, 498–562	ERG	65
	*Lethrus apterus*	355, 525	ERG	49
	*Protaetia brevitarsis*	360–380, 510–530	ERG	57
Tenebrionidae	*Tenebrio molitor*	520–550	ERG	47

*ERG* electroretinogram, *IntCell* intracellular recording

**Fig. 1:**
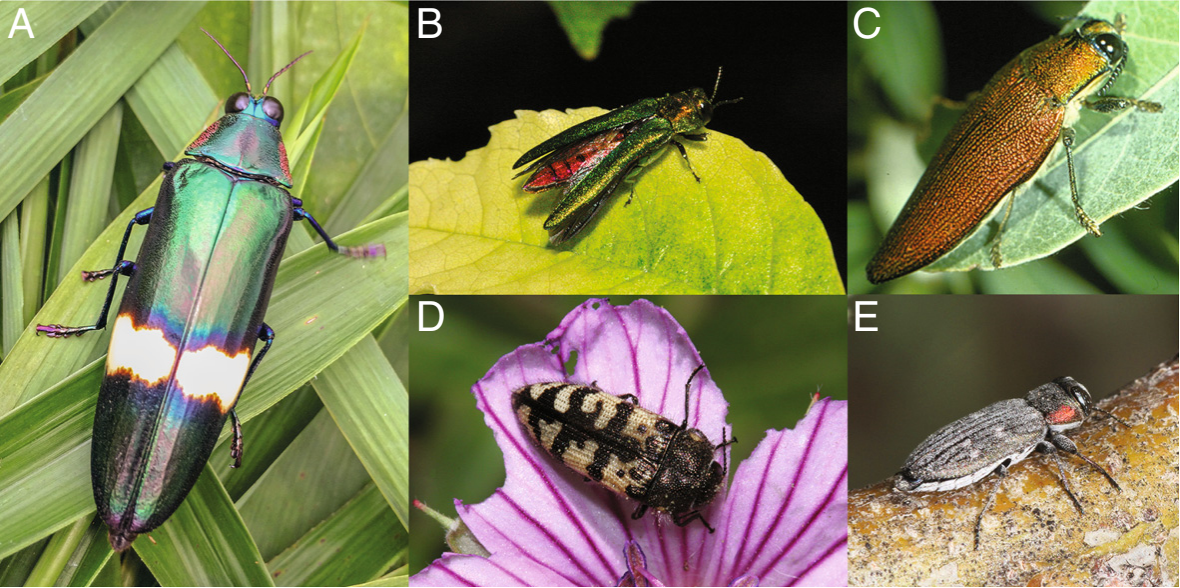
Jewel Beetles (Coleoptera: Buprestidae) sequenced in this study. **a** *Chrysochroa tonkinensis* (Descarpentries); **b** *Agrilus planipennis* Fairmaire (EAB), dorsal view, exhibiting “wing flashing”; **c** *Steraspis amplipennis* (Fåhraeus); **d** *Acmaeodera diffusa* Barr; **e** *Chrysobothris lateralis* Waterhouse. Photo credit: **a** NPL; **b** JPL; **c** Charles Bellamy, Sacramento, CA; **d** Nicky Davis; **e** Lon Brehmer and Enriqueta Flores-Guevara, Redington, AZ

The jewel beetles (Buprestidae) are an ideal candidate for studying potential molecular complexity within beetles. Most members of the group are diurnally-active, highly visual, and display impressive patterns of metallic and pigmented coloration (e.g., *Chrysochroa*, *Acmaeodera*, Fig. 1) that are a central signal to mate recognition [58, 67–69]. Furthermore, ERG data suggest the economically important *Agrilus planipennis* Fairmaire (emerald ash borer—EAB; Fig. 1c) has multiple photoreceptor sensitivities to UV, violet, SW and LW portions of the spectrum [58]. Females also exhibit additional sensitivity to longer wavelengths (640–670 nm).

### Objectives

The purpose of this work is to investigate the molecular basis of potential short-wavelength sensitivity in members of the beetle family Buprestidae. The objectives of this study are to: 1) investigate the presence/absence of the SWS opsin class, 2) identify potential duplications within UVS and LWS opsin classes, and 3) examine opsin protein/chromophore interactions to identify amino acid substitutions that could confer shifts towards short-wavelength sensitivity.

## Results and discussion

### Buprestidae opsin classes and copies

**Fig. 2:**
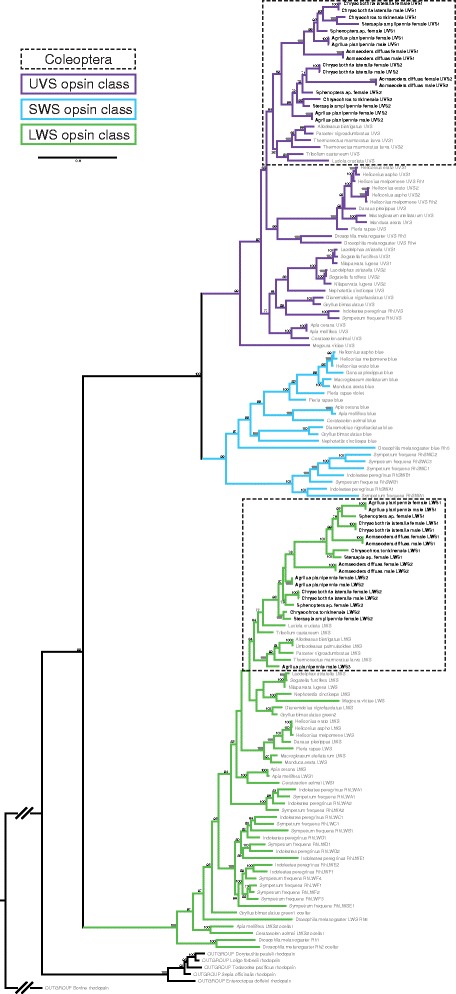
Opsin gene topology of 145 sequences based on the single best ML tree under the BIC best-fit protein model LG + F + I + G4, LogL = -39087.214. UFBootstrap values based on 10,000 replicates are given at nodes (UFBootstrap values < 50 not shown). Tree was rooted to bovine rhodopsin outgroup. Jewel beetle taxa are bolded

**Fig. 3:**
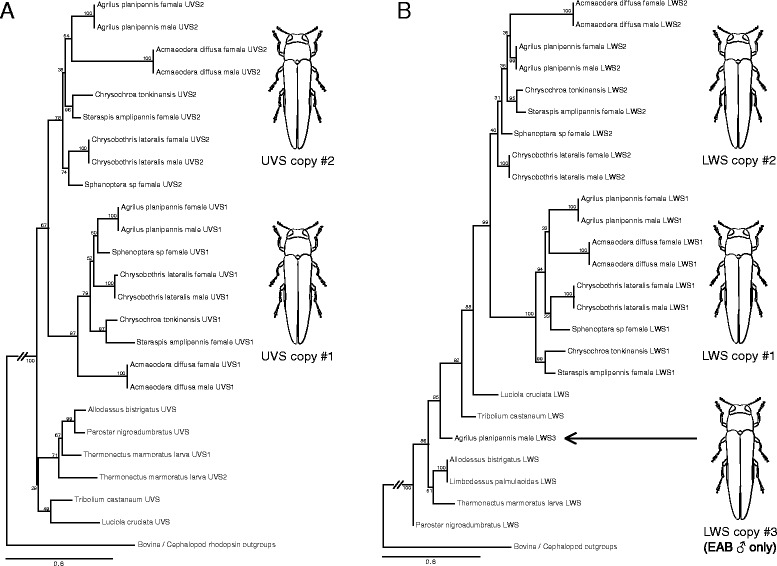
**a** Coleoptera UVS opsin gene topology based on the single best ML tree under the BIC best-fit protein model LG + F + I + G4, LogL = -8783.692. UFBootstrap values based on 10,000 replicates are given at nodes (UFBootstrap values < 50 not shown). Tree was rooted to bovine and cephalopod rhodopsin outgroups. Jewel beetle taxa are bolded. **b** Coleoptera LWS opsin gene topology based on the single best ML tree under the BIC best-fit protein model LG + F + I + G4, LogL = -8270.226. UFBootstrap values based on 10,000 replicates are given at nodes. Tree was rooted to bovine and cephalopod rhodopsin outgroups. Jewel beetle taxa are bolded

The lack of a SWS opsin class in beetles is somewhat unexpected, as the diversity of eye morphologies and visually-mediated behaviors within the group [64, 68, 70–72] would suggest sensitivity to the full spectral range of visible light. Within the Buprestidae, although previous physiology data supports sensitivity of one buprestid (EAB) to short wavelengths [58], we did not recover a SWS (blue) opsin class in any of the taxa analysed. However, we detected at least four opsin copies in all buprestids (Figs. 2 and 3a-b)—two UVS and two LWS opsins, the most detected in any beetle species. In the male EAB, a partial third LWS opsin copy (containing all seven trans-membrane domains) was detected.

The opsin topology generated for all taxa (Fig. 2) recovered well-supported clades (UFBoot ≥99) for insect UVS, SWS, and LWS opsin classes. The buprestid UVS1 + 2 clade was recovered as sister to the remaining Coleoptera UVS in both the full taxon topology (Fig. 2) and Coleoptera-specific UVS topology (Fig. 3a). In both analyses, the buprestid UVS1 and UVS2 clades were recovered with high support (UFBoots >75) and sister to one another. The buprestid LWS clade was recovered as nested within the other coleopteran LWS opsins (UFBoot ≥ 98 in full and Coleoptera-specific LWS topologies), with the exception of the male EAB LWS3, which was recovered as sister to the diving beetles in the full topology (Fig. 2), or nested within the beetles in the Coleoptera-specific LWS topology (Fig. 3b). The buprestid LWS1 clade was recovered with high support in both analyses (UFBoot ≥ 99) and as sister to the LWS2 buprestid paralogs (or opsin copies). One notable difference between the full taxon LWS topology and the Coleoptera-specific LWS topology is the placement of *Acmaeodera diffusa* Barr LWS2, which is recovered either as sister to buprestid LWS1 opsins (full taxon topology, Fig. 2), or nested within the remaining buprestid LWS2. This is likely due to the sequence dissimilarity of *Acmaeodera diffusa* from the other buprestid taxa, resulting in long branches. Interestingly, members of *Acameodera* generally possess pigmented coloration, and mate recognition is thought to occur on the flowers on which they feed. This is in contrast with the predominant iridescent coloration and mate-seeking behavior of most other buprestids.

**Fig. 4:**
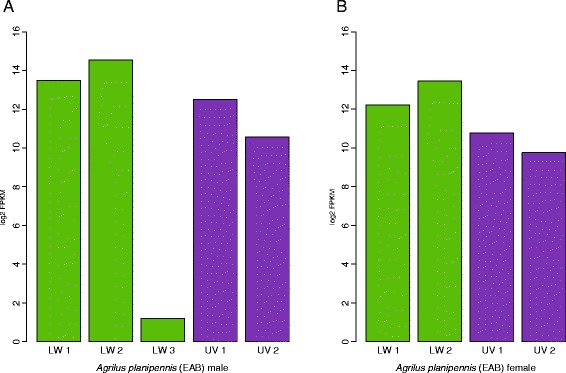
Emerald ash borer opsin gene copy expression trends: **a** EAB male; **b** EAB female

The presence of a third LWS opsin copy (LWS3) in male EAB only and the recovery of this opsin outside of the Buprestidae LWS clade in both full taxon and Coleoptera-specific analyses are of interest. Tests of opsin copy relative expression from the generated EAB male and female transcriptomes yielded comparable expression levels of LWS1 and LWS2 between copies and sexes, as did levels of UVS1 and UVS2 opsins. LWS3, however, was expressed at significantly lower levels in the male EAB (Fig. 4). Although the presence of a third male LWS opsin may have some biological relevance in male-driven EAB mate recognition strategies, discrepancies in data suggest this opsin is more likely to be residual expression from the larval life stage rather than a unique adult copy. Physiological data from EAB suggests a broader range of spectral sensitivities recorded in EAB females [58] as opposed to the copy-rich males, and the lower expression levels of LWS3 in relation to LWS1&2 (Fig. 4) suggest rarity within the transcriptome. Phylogenetically, the lack of LWS3 recovery in any other buprestid taxa and molecular similarity of EAB LWS3 to a larval diving beetle and other Coleoptera opsins further support this third copy as a potential larval hold-over (Fig. 2, Additional file 1: Table S3). Confirmation of this hypothesis will require the generation of additional transcriptomes across life stages to accurately characterize the EAB male LWS3 opsin. Nevertheless, the loss of the SWS class combined with UVS and LWS opsin duplications across all buprestid taxa indicates a functional significance for opsin variability within the highly visual jewel beetles.

### Amino acid comparisons of opsin genes

Intra- and interspecific pairwise comparisons of Buprestidae across the opsin sequences for amino acid similarity, as well as comparisons of opsin paralogs in other insect taxa, are given in Additional file 1: Table S3. In general, amino acid sequence similarity between paralogous opsin copies within the buprestid individuals (calculated as the number of both identical and chemically conserved amino acids divided by total amino acid number), is noticably lower than the ranges recovered in other insects with duplications (e.g., UVS1/UVS2 similiarity in *Acmaeodera diffusa*: 72 %; all other Buprestidae: 82–84 %; *Heliconius* spp.: 93–94 %, Hemiptera spp.: 91–92 %). Within Buprestidae, opsin copies were more similar to interspecific orthologs (e.g., LWS2 opsin copies between buprestid species: 87–95 % similarity) than to intraspecific paralogs (e.g., *Acmaeodera diffusa* opsin UVS1/UVS2, LWS1/LWS2 paralogs: 72 and 75 %, respectively; all other buprestid paralogs 78–86 %). The LWS3 opsin in male EAB is more similar to the LWS opsins of the larval *Thermonectus marmoratus* and all other adult beetles (86–93 % similarity) than to the EAB LWS1 and LWS2 paralogs (78 and 86 % similarity, respectively). This degree of sequence divergence suggests that opsins have undergone greater diversification within buprestids than in other insect taxa.

### Opsin structure and ligand binding amino acid variations

Sequence data for the recovered buprestid opsins provided us the opportunity to examine potential similarities in opsin structure and spectral sensitivities with other insects. Homology-based modeling resulted in best-fit model predictions utilizing squid rhodopsin (*Todarodes pacificus* (Steenstrup), RSCB protein databank: 2Z73; [15]) as the template for all opsins recovered from the generated transcriptomes. To confer short-wave sensitivity in buprestids, we predict that substitutions in the UVS duplicates cause a bathochromic (toward a longer λ) shift in spectral sensitvity, and/or substitutions in the LWS duplicates cause a hypsochromic (toward a shorter λ) shift of the visual pigment. To investigate this possibility, we performed tests of positive selection (PS) and analyzed the amino acid composition and structure of the chromophore binding pocket in the buprestid opsins (Figs. 6 and 7, Tables 2 and 3).

**Table 2:** Candidate residues for spectral shifting between paralogous UVS opsin copies e.g., UVS1 vs. UVS2) based on combined evidence

Gene	Site	Location	Positive branch-site selection in Buprestidae (this study)	Branch label (see Fig. X)	UVS1 residue	UVS2 residue	Structural significance of substitution (this study)	Positive selection in other organisms at homologous sites (previous studies)	Wavelength shifts in other organisms at homologous sites (previous studies)
UV	12	EL	*Acmaeodera diffusa* UVS2	UV-F	V	P	imposed backbone rigidity	Lepidoptera: *Heliconius* UV A37E [28]	-
	105	EL	Buprestidae UVS1	UV-C	T	T/L	gain of polarity (T–L)	Lepidoptera: *Heliconius* UV T121I [28]	-
			*Acmaeodera diffusa* UVS2	UV-F	A	L	increase in size		
	107	EL	Buprestidae UVS2	UV-D	H	F/M/Y	loss of positive charge (to polar or neutral), increase in size (H–Y)	Lepidoptera: *Heliconius* UV A123S/T [28]	-
	118	CBP, TM3	-	-	T/S	T	most likely insignificant	Coleoptera: *Photinus* UVS S133 [43]	Lepidoptera: Pieris rapae SWS S116A (13 nm hypsochromic shift) [11]
	125	TM3	*Acmaeodera diffusa* UVS2	UV-F	G	S	gain of polarity, increase in size	-	Vertebrates: Rh1 125 (-5 nm to +8 nm shifts) [75]
	137	CL	Buprestidae UVS2	UV-D	T	H	gain of positive charge (from polar), increase in size	-	Mammals: Rhodopsin V137M (retinitis pigmentosa) [76]
	172	TM4	*Acmaeodera diffusa* UVS2	UV-F	F	Y	gain of polarity	-	Lepidoptera: LWS L145M/F/I (blue shifts) [78]
	186	CBP, EL	Buprestidae UVS2	UV-D	A	Q	gain of polarity, increase in size	Lepidoptera: *Heliconius* UVS S202A [28]	-
	188	CBP, EL	*Acmaeodera diffusa* UVS2	UV-F	S	G	loss of polarity, decrease in size	Lepidoptera: *Heliconius* UVS T204S [28]	-
	207	CBP, TM5	Buprestidae UVS2	UV-D	I	L	most likely insignificant	-	Vertebrates: SWS L207M/I (6 nm shift plus additive effects) [82, 83]
	242^a^	CL	Buprestidae UVS2	UV-D	A	Q	gain of polarity, increase in size	Coleoptera: *Photinus* UVS A268 [43]	-
					T/S	Q	change in polarity, increase in size		
	261	CBP, TM6	-	-	F/Y	F	loss of polarity (Y–F)	-	Human: Y277F (red/green pigment variation) [81]; Vertebrates: SWS T261F (5 nm blue shift) [82]
	272	TM6	*Acmaeodera diffusa* UVS2	UV-F	S	C	gain of potential for disulfide bonding	Coleoptera: *Photinus* UVS S299 [43]	-
	293	TM7	*Acmaeodera diffusa* UVS2	UV-F	C	L	loss of polarity and potential for disulfide bonding	Lepidoptera: *Heliconius* UVS V321I [28]	Human: Y309F (red/green pigment variation) [81]
	294	TM7	Buprestidae UVS1	UV-C	F	T/A	loss of polarity (F–T), increase in size	Coleoptera: *Photinus* UVS T321 [43]	-
			*Acmaeodera diffusa* UVS2	UV-F	C	F	increase in size, loss of polarity and potential for disulfide bonding		

^a^gap between bovine reside 242 & 243, original residue A267 in *Acmaeodera diffusa* UVS1Residues provided are for the lineage in which positive selection occurs. Sites numbered according to bovine in column two; sites numbered according to original study in columns 9–10. CBP: chromophore binding pocket; CL: cytoplasmic loop; EL: extracellular loop; TM: transmembrane helix

**Table 3:** Candidate residues for spectral shifting between paralogous LWS opsin copies e.g., LWS1 vs. LWS2) based on combined evidence

Gene	Site	Location	Positive branch-site selection in Buprestidae (this study)	Branch label (see Fig. X)	LWS1 residue	LWS2 residue	LWS3 residue (EAB males)	Structural significance of substitution (this study)	Positive selection in other organisms at homologous sites (previous studies)	Wavelength shifts in other organisms at homologous sites (previous studies)
LW	44	TM1			I	I/M		most likely insignificant (I–M)	Lepidoptera: *Limenitis* spp. LWS I17M (positive selection and blue shifts) [21, 78]; I44M suggested for Lepidoptera [77]; Mammals: Rhodopsin M44T (3 nm blue shift) [76]	
	46	TM1	Buprestidae LWS1	LW-E	T	V		gain of polarity		Vertebrates: SWS F46A/L [79, 83]
					I	V		most likely insignificant		
					C	I		gain of polarity and potential for disulfide bonding		
	91	TM2			A	V/S		most likely insignificant (A–V) or gain of polarity (A–S)	Lepidoptera: *Limenitis* spp. LWS A64S (positive selection and blue shifts) [21, 78]; Vertebrates: SWS P91S (10 nm blue shift) [82]	
	93	TM2	*Acmaeodera diffusa* LWS2	LW-F	P	E		gain of backbone flexibility, increase in size, gain of negative charge (from neutral)		Vertebrates: SWS T93L/V [74, 83]
	122	CBP, TM3			T/C	C		gain of potential for disulfide bonding (T–C)		Bovine: E122Q (17 nm blue shift) [12]; Vertebrates: SWS I122M (6 nm blue shift) [82]
	123	TM3	Coleoptera LWS (some) + *Agrilus planipennis* LWS3 (male)	LW-H	V	T	I	loss of polarity (T–I) or most likely insignificant (V–I)		Lepidoptera: LW 97 [8, 20]
			Buprestidae LWS1	LW-E	V/A	T		loss of polarity		
	156	TM4	Coleoptera LWS (some) + *Agrilus planipennis* LWS3 (male)	LW-H	W	R	Q	gain of positive charge from neutral (W–R), gain of polarity (W–Q), loss of positive charge (to polar) and decrease in size (R–Q)	Lepidoptera: *Heliconius* UVS M171L [28]	
			*Acmaeodera diffusa* LWS 1	LW-G	W	R		loss of positive charge (to neutral)		
	164	TM4			S/T	S/T/C/A		most likely insignificant (S/T–S/T)	Lepidoptera: *Limenitis* spp., LWS S137A (positive selection and blue shifts) [21]; S138A suggested for Lepidoptera [77]; Humans: S180A (red/green pigment variation, 5 nm shift) [73, 81]	
								gain of potential for disulfide bonding (S/T–C)		
								loss of polarity (S/T–A)		
	170	TM4	Coleoptera LWS (some) + *Agrilus planipennis* LWS3 (male)	LW-H	A	A	L	increase in size (A–L)	Lepidoptera: *Heliconius* UVS M185L [28]	
	197	EL	Coleoptera LWS (some) + *Agrilus planipennis* LWS3 (male)	LW-H	D	D	E	most likely insignificant (D–E)		Lepidoptera: LWS 170 (blue shifts) [78]; Vertebrates: LWS 197 [80]
	211	CBP, TM5	Buprestidae LWS1	LW-E	C	V		gain of polarity and disulfide bond potential	Lepidoptera: *Heliconius* UVS S125T/A [28]	Bovine: Rh1 H211C (5 nm shift) [12]; Vertebrates: SWS S211C (2 nm blue shift) [82]
	242^a^	CL	*Acmaeodera diffusa* LWS1	LW-G	A^b^	A		no substitution^b^	Coleoptera: *Photinus* UVS A268 [43]	
	269	CBP, TM6			L/A/M	L		increase in size (A–L)		Bovine: LWS A269T (14 nm toward red) [9]; Human: T269A (red/green pigment variation, 15 nm shift) [73, 81]
								most likely insignificant (M–L)		
	274	TM6	Buprestidae LWS1 + *Acmaeodera diffusa* LWS2	LW-C	T/V/L/A	T		loss of polarity (V/L/A–T)		Lepidoptera: LWS 259 [20]
	281	EL	Coleoptera LWS (some) + *Agrilus planipennis* LWS3 (male)	LW-H	A	A	K	gain of positive charge (from neutral) and increase in size (A–K)	Coleoptera: *Photinus* LWS T309 [43]	

^a^gap between bovine reside 242 & 243, original residue A268 in *Acmaeodera diffusa* LWS1

^b^although there is no variation of amino acids at site A268 in *Acmaeodera diffusa*, all other buprestids Residues provided are for the lineage in which positive selection occurs. Sites numbered according to bovine in column 2; sites numbered according to original study in columns 9–10. CBP: chromophore binding pocket; CL: cytoplasmic loop; EL: extracellular loop; TM: transmembrane helix

### Tests of Positive Selection (PS)

**Fig. 5:**
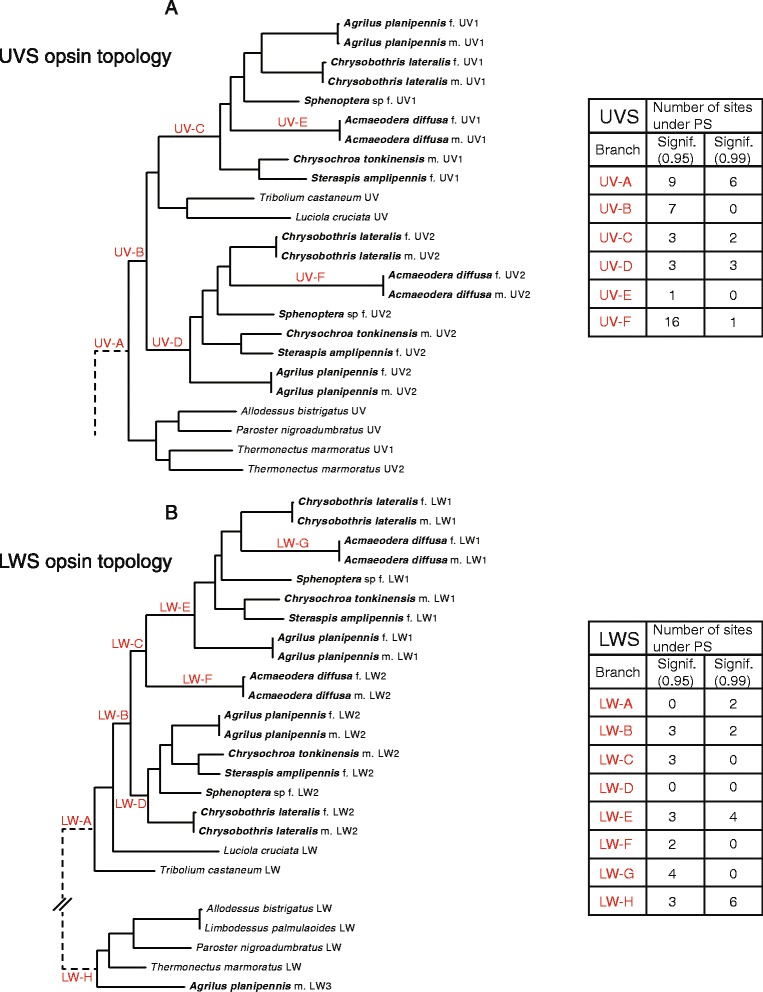
**a** UVS opsin topology with tests of branch-site positive selection. Branch labels are in red. Numbers of amino acid sites under positive selection for p-values of 0.95 and 0.99 are provided in the table insert. For full listing of amino acid sites under selection and statistics, see Additional file 2: Table S4 and Additional file 3: Table S5. **b** LWS opsin topology with tests of branch-site positive selection. Branch labels are in red. Numbers of amino acid sites under positive selection for p-values of 0.95 and 0.99 are provided in the table insert. For full listing of amino acid sites under selection and statistics, see Additional file 2: Table S4 and Additional file 3: Table S5

A list of all sites recovered as under PS from the lineages tested in our analyses (Fig. 5a-b), as well as the homologous site across other opsin copies within those taxa is presented in Additional file 2: Table S4. Log-likelihood values and parameter estimates of the branch-site tests of PS are provided in Additional file 3: Table S5. Only sites in UVS/LWS clades under PS that exhibit significant amino acid structural variation and/or additional supporting data will be discussed below.

### Amino acid substitutions of importance

**Fig. 6:**
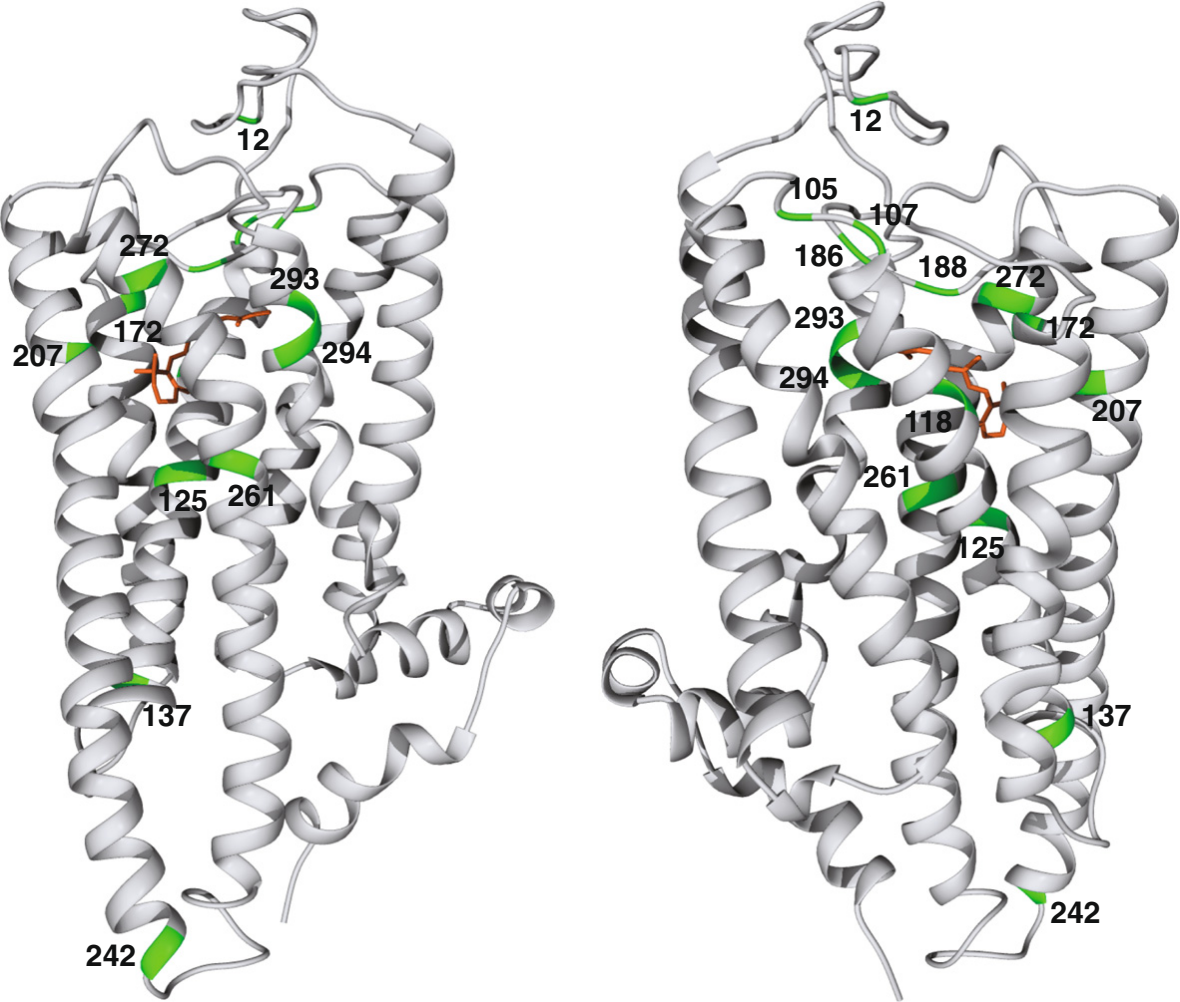
3D model of *Acmaeodera diffusa* UVS2, two views shown (180° rotation). Retinal is in orange. Residues in *green* are those reported as potentially significant to spectral tuning in Buprestidae, as listed in Table 2. Numbering is according to bovine rhodopsin

**Fig. 7:**
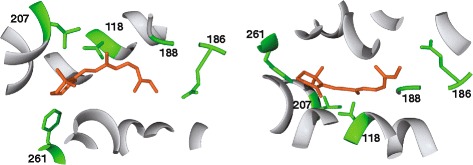
Chromophore binding pocket of *Acmaeodera diffusa* UVS2, two views shown. Retinal is in *orange*. The residues shown comprise the chromophore binding pocket, as predicted by COACH (within 4 angstroms of retinal). Residues and their side chains in green are those reported as potentially significant to spectral tuning in Buprestidae, as listed in Table 2. Numbering is according to bovine rhodopsin

An analysis of residue substitutions in the buprestid opsin copies identified points of potential bathochromic or hypsochromic spectral variation in the copies. Of particular interest are substitutions that are: 1) under PS in one opsin paralog only, 2) similar to substitutions found in other taxa at homologous sites that confer a change in spectral sensitivity [73–80], or 3) present within UVS or LWS copies and introduce a residue that significantly alters the chemical nature or shape of the chromophore binding pocket (see Figs. 6 and 7). Amino acid substitutions that meet several structural critera, as defined below, are presented in Tables 2 and 3. An increase or decrease in size indicates the length of the amino acid side chain differs by more than one (non-H) atom. Such changes have the potential to alter helical packing and orientation by their steric variation. A gain of polarity or charge confers the ability for hydrogen bonding, which can stabilize structure and also is important in the chromophore binding pocket for light-induced activation. Likewise, a loss of polarity or charge removes the ability to hydrogen bond and creates a hydrophobic region.

In Buprestidae UVS copies, there are 15 residues that meet these criteria and hence have the potential for bathochromic tuning toward blue sensitivity (Table 2). Of these, the A186Q and Y261F substitutions are significant because they change polar residues in the binding pocket, which may alter the H-bonding that occurs in an extended network from the protonated Schiff base, and subsequently the activation of retinal. Site 186 was recovered as being under PS in the Buprestidae UVS2 opsin clade (branch “UV-D” in Fig. 5a), although 261 was not. These two substitutions are also present in the butterfly species *Heliconius*, which has two UVS copies (see Table 2) and a unique spectral sensitivity reported for each copy [28]. Additionally, Y261F has been identified in humans as one of two residues responsible for spectral tuning between red and green wavelengths in a hypsochromic direction [81].

An I-L substitution between Buprestidae UVS1 and UVS2 at binding pocket site 207 was found to be under PS in the UVS2 clade (Table 2; branch “UV-D” in Fig. 5a), and although this substitution introduces only minimal steric variation to the binding pocket, a homologous substitution in vertebrates results in a 6 nm shift in the SWS opsin, with additive effects on spectral shifting when in combination with other substitutions [82, 83]. A S118T binding pocket substitution in Buprestidae UVS paralogs does not exhibit significant structural variation, nor was this substitution recovered as under PS, but substitutions at this homologous site exhibited PS in a lampyrid [43], and a 13 nm hypsochromic shift in a lepidopteran [11]. Three additional non-binding pocket sites with substitutions in Buprestidae UVS2 opsins—107, 137, and 242—were recovered as being under positive selection, exhibit some degree of significant structural variation, and are implicated in spectral shifting or under PS in other organisms (see Table 2). There are a number of additional substitutions within the Buprestidae UVS1 clade (site 105, 294; branch “UV-C” in Fig. 5a) or *Acmaeodera diffusa* UVS2 clade (site 12, 105, 125, 172, 188, 272, 293, and 294; branch “UV-F” in Fig. 5a) that are under PS, exhibit significant structral changes, and/or are recovered as important in other taxa, but are not located in the binding pocket or are invariant in all Buprestidae UVS opsin paralogs. These residues can be found in Table 2 and have been mapped on an opsin reconstruction (Figs. 6 and 7).

In Buprestidae LWS copies, there are 15 residues with the potential for bathochromic tuning toward SW sensitivity (Table 3), although the correlations between specific paralogs (e.g., LWS1 vs LWS2) are less clear than in the UVS opsin copies. Three substitutions are present in the binding pocket and exhibit some degree of significant structural change (C122T, V211C, and A/ML269L), but only site 211 was recovered as under PS in Buprestidae LWS1 (branch “LW-E” in Fig. 5b) and under PS in another insect group (Lepidoptera: *Heliconius*; [28]). All three sites, however, are implicated in 2–17 nm wavelength shifts in vertebrates [12, 82]. Sites 46, 123, and 274 are located outside the chromophore binding pocket, but exhibit substitutions of structural significance, are recovered as under PS in Buprestidae UVS1 copies (branches “LW-C” and “LW-E” in Fig. 5b), and are implicated in wavelength shifts at homologous sites in Lepidoptera LWS opsins [8, 20] and vertebrate SWS opsins [79, 83]. Although substitutions at sites 44, 91, and 164 were shown to be under PS and implicated in several independent “blue” shifts in *Limenitis* butterflies [21, 77, 78] and vertebrates [73, 76, 81, 82], these sites were not recovered as under PS or possessing substitutions of significant structural variation in Buprestidae, although additive effects on spectral tuning cannot be discounted. Several other sites were recovered under PS and/or with significant structural variation in LWS opsin copies of specific buprestid taxa (93, 156, 242, 274 in *Acmaeodera diffusa*; 123, 156, 170, 197, 281 in male *Agrilus planipennis* LWS3), but variation was not present across all Buprestidae LWS opsin paralogs (Table 3).

In summary, our UVS1/UVS2 buprestid copies possess five sites with amino acid substitutions in the chromophore binding pocket (118, 186, 188, 207, 261) and five sites outside the binding pocket (105, 107, 137, 242, 294) that are specific to buprestid UVS paralogs and are strongly implicated in bathochromic spectral tuning in other organisms (Table 2). Three buprestid LWS1/LWS2 sites in the chromophore binding pocket (122, 211, 269) and three sites outside the binding pocket (46, 123, 274) suggest hypsochromic shifts and are recovered in other organisms (Table 3). Note that many of these are reported to contribute to spectral tuning in other organisms and could do so through translation of structural variation to either the chromophore binding pocket or the intracellular face that binds signaling proteins. Taken collectively, we provide evidence for a number of candidate sites that might enable short-wavelength sensitivity among Buprestidae, which is specifically provided for by the duplication of UVS and/or LWS opsin copies.

## Conclusions

This study reveals previously unseen molecular complexity underpinning spectral sensitivity within Coleoptera. Previous electrophysiological work on the emerald ash borer (*Agrilus planipennis* Fairmaire) demonstrated a greater diversity in photoreceptor sensitivity (UV, SW and LW) than the vast majority of other beetles (UV and LW). Our results suggest that, in the absence of a SWS opsin class, sensitivity has been gained through subsequent shifts in spectral sensitivity (spectral tuning) of UVS/LWS opsin duplications, achieved by specific amino acid substitutions within the opsin proteins. Our analysis of potential spectral tuning sites within these copies highlights a number of substitutions that are likely to have conferred SW sensitivity within these species. This study forms the basis for future site-specific mutagenesis of the non-conserved substitutions to definitively confirm these as sites critical to spectal shifting within buprestids.

While other groups of beetles with a well-established reliance on visual cues (e.g., Lampyridae) do not appear to possess a SWS opsin or duplications of the LWS/UVS, utility of and reliance on filtering and screening pigments is likely to achieve spectral shifting. Both the jewel beetles and fireflies are highly visual coleopterans, although the activity periods (diurnal vs. crepuscular/nocturnal), light environments, and natural histories (fine-tuning to specific wavelengths in Lampyridae) suggest fundamentally different visual systems between members of the two families. It is possible that filtering pigments play a shifting role in Buprestidae as well, although the expanded spectral sensitivity recorded for *Agrilus planipennis*, combined with the ubiquitous opsin duplication and homologous copy similarity, strongly suggest a function of biological relevance beyond filtering pigments to achieve sensitivity in a missing middle-wavelength opsin class. This work confirms that UVS/LWS opsin duplications and amino acid substitutions are widespread within Buprestidae, and the putative gained expansion in spectral discrimination is likely highly advantageous for a group that relies so heavily on visual cues for mate and host selection.

## Methods

### Taxa sequenced

Nine specimens from five species, representing three male/female pairs were selected for sequencing (Fig. 1, Additional file 4: Table S1). Four of the six currently-recognized subfamilies of Buprestidae are represented, providing sufficient phylogenetic coverage to explore opsin diversity across the group. A male and female pair of the following taxa were sequenced: *Acmaeodera diffusa* Barr (subfamily Polycestinae), *Agrilus planipennis* Fairmaire (emerald ash borer—EAB; subfamily Agrilinae), and *Chrysobothris lateralis* Waterhouse (subfamily Buprestinae). Single specimens of three chrysochoines, *Chrysochroa tonkinensis* (Descarpentries) (male), *Steraspis amplipennis* (Fåhraeus) (female), and *Sphenoptera* sp. (female) were also sequenced. These selected species represent extreme variations in size, habitat, natural history, and phylogenetic placement, allowing for a first look at the opsin diversity within the Buprestidae. Male and female EAB specimens were obtained from the USDA EAB Rearing Facility by JPL in Brighton, MI, USA. The specimen of *Chrysochroa tonkinensis* was collected in northern Vietnam and the specimen if *Steraspis* in Rwanda by NPL and SMB. NPL collected the male/female pairs of *Acmaeodera diffusa* and *Chrysobothris lateralis* were collected in UT and NM, respectively. In an attempt to capture maximum opsin expression, all specimens were collected and processed during typical daylight activity and flight hours (~10:00–14:00 h). The heads were disarticulated from live specimens, split longitudinally with a sterile razor blade, and submerged in a RNA*later*® (ThermoFisher Scientific) solution (protocol in [84]). The specimens were stored at -80 °C until RNA extraction.

### Vouchers

Total RNA-extract and the remainder of specimens used in this study (thorax and abdomen) are deposited in the Insect Frozen Tissue collection at Brigham Young University (BYU, Provo, UT, USA).

### Molecular data

***Transcriptomics***: Total RNA was extracted from the eyes of each individual using NucleoSpin RNA II isolation extraction kits (Clontech) and reverse-transcribed into cDNA libraries using the Illumina TruSeq RNA v2 sample preparation kit. The prepared mRNA libraries were sequenced on an Illumina HiSeq 2000 utilizing 101-cycle paired-end reads by the Microarray and Genomic Analysis Core Facility at the Huntsman Cancer Institute at the University of Utah (Salt Lake City, UT, USA). ***Transcriptome Assembly***: Quality control, assembly, and transcriptome analysis to facilitate downstream phylogenetic analyses were performed using existing computational tools (see below) combined into a pipeline in the Bybee Lab (BYU). RNA-seq reads were trimmed using the Mott algorithm implemented in PoPoolation [85], with a minimum read length = 40 and quality threshold = 20. The de novo assembly of the transcriptome contigs was carried out using Trinity [86] under the default parameters. Results from the buprestid transcriptome assemblies are summarized in Additional file 5: Table S2. ***Opsin Genes***: Potential light-interacting genes were isolated from each transcriptome by utilizing the Phylogenetically-Informed Annotation (PIA) tool [87], implemented in Galaxy ([88–90]). To identify putative opsins, all individual contigs isolated by the PIA tool were BLASTed, as implemented in Geneious® R6, v.6.1.8 (http://www.geneious.com, [91]) utilizing the “nr” database option (searching GenBank, RefSeq, EMBL, DDBJ, and PDB databases) and the BLASTN algorithm set to 100 maximum hits and default E-value threshold of 0.001. Similar hits were then assessed for E-value and sequence type/description. Opsin sequences were deposited in GenBank (see Additional file 4: Table S1).

### Phylogenetic reconstruction

In order to predict the evolutionary relationships of opsin gene sequences generated from the transcriptomes, additional opsin data from other taxa across Insecta were downloaded from GenBank (see Additional file 4: Table S1 for accession numbers) for the construction of an opsin topology. Including the data generated for this study, 139 opsin sequences from 35 taxa across seven orders of insects were included in the analysis. Additionally, bovine rhodopsin and five cephalopod rhodopsin sequences were selected as outgroups based on the phylogenetic relationships of opsins recovered by Porter et al. [92]. In addition to GenBank, data for *Drosophila melanogaster* were obtained from FlyBase [93]. Opsin genes were restricted to the CDS by manually trimming untranslated regions (UTRs) for each sequence in Geneious®. All opsin sequences were then checked for open reading frames, translated to amino acids, and aligned with MAFFT v.7.017 [94] under the “Auto” strategy as implemented in Geneious® R6 (BLOSUM62 scoring matrix, 1.53 gap open penalty, 0.123 offset value). Additional alignments of strictly UVS and strictly LWS opsins from only Coleoptera plus six outgroups were generated as above. All three alignments are available in the Dryad Digital Repository (doi:10.5061/dryad.f8584). Using protein alignment and model-testing options within both IQ-Tree [95] and PartitionFinder v.1.1.1 [96, 97] the LG + I + G4 + F model was determined to be the most probable amino acid substitution model for all three alignments. This model was used to perform independent ML tree searches in IQ-Tree with 10,000 ultrafast bootstrap iterations (UFBoot; [98]) to assess the nodal support. Each tree search was repeated 1000 times in order to increase the chance of recovering the most likely topology with the highest log-likelihood value (LogL). All the aformentioned analyses were conducted using the resources of the Fulton Supercomputing Lab at Brigham Young University. Trees were visualized in Figtree v.1.4.2 (Rambaut, Andrew. “FigTree.” http://tree.bio.ed.ac.uk/software/figtree/), and tree figures were constructed in Adobe Illustrator CC 2014.

### Expression trends

Expression of each opsin copy in FPKM (fragments per kilobase of transcript per million mapped reads) for *Agrilus planipennis* was calculated using the algorithm of abundance estimation implemented in RNA-seq by Expectation-Maximization (RSEM, [99]).

### Tests of positive selection

Nucleic acid opsin sequences were first aligned with MAFFT v.7.017 [94] under the “Auto” strategy as implemented in Geneious® R6 (BLOSUM62 scoring matrix, 1.53 gap open penalty, 0.123 offset value). Taxa were restricted to insects (see Additional file 4: Table S1), and the alignment is available in the Dryad Digital Repository (http://dx.doi.org/10.5061/dryad.f8584) Tests for possible episodic positive selection operating on opsins were performed in PAML v4 [100]. Using branch-site new model A, we tested the ancestral UVS and/or LWS branches and their sites for positive selection across and between several lineages (see Fig. 5a-b, Additional file 2: Table S4 and Additional file 3: Table S5) of Buprestidae and other Coleoptera. The log-likelihood of each competing model was compared against the null model of fixed ω = 1 (no selection) with the Likelihood Ratio Test (LRT) using *χ*^2^ distributions with appropriate degrees of freedom. To avoid model trapping in a local optimum, we ran analyses at least three times specifying initial ω values at 0.1, 1 and 2. Then Bayes empirical Bayes (BEB) [101] procedure was used to calculate posterior probabilities for the site classes.

### Amino acid composition and opsin structure

To determine amino acid variation, opsin sequences were translated and compared intra- and inter-specifically in Geneious® under the BLOSUM62 score matrix [102, 103], with a similarity threshold of 1 (Additional file 1: Table S3). Trans-membrane helices were identified for all Coleoptera included in the analyses using TM-Coffee on the T-Coffee web server under default settings [104]. Sites of amino acid variability were analyzed across and within insect species (Tables 2 and 3) to determine the potential for spectral tuning based on alignment and amino acid chemical nature. Homology-based structural modeling was performed via the I-TASSER server [105–107], including concurrent protein-ligand binding site predictions by COACH [108, 109]. In order to draw comparisons of opsin structure and variation and across the class, additional modeling was performed on selected insect opsin proteins (Figs. 6 and 7). Models with the highest c-score were used in further structure analysis conducted with UCSF Chimera [110].

### Consent for publication

Not applicable.

### Availability of data and material

The datasets supporting the results of this article are available in the Dryad Digital Repository (http://dx.doi.org/10.5061/dryad.f8584) [111]. Nucleic acid sequence data supporting the results of this article are available in the NCBI GenBank Repository (http://www.ncbi.nlm.nih.gov/genbank/). Accession numbers are available in Additional file 4: Table S1.

## Additional files

Additional file 1: Table S3. Amino acid sequence comparison of opsin gene copies for selected taxa. (PDF 110 kb) Additional file 2: Table S4. Branches and sites under positive selection. *P* = 95–99. If bolded, P= >99. Amino acid residues are numbered according to bovine rhodopsin. (PDF 176 kb) Additional file 3: Table S5. Log-likelihood values and parameter estimates of branch-site tests of positive selection. (PDF 117 kb) Additional file 4: Table S1. Taxon sampling data and GenBank accession numbers (http://www.ncbi.nlm.nih.gov) for gene sequences analyzed in this study. (PDF 128 kb) Additional file 5: Table S2. Statistics for transcriptomes generated in this study. (PDF 91 kb)

## Abbreviations

CDS: coding sequence
EAB: emerald ash borer, *Agrilus planipennis* Fairmaire (Coleoptera: Buprestidae: Agrilinae)
ERG: electroretinogram
LWS: long-wavelength sensitive
NGS: next-generation sequencing
PS: positive selection
SWS: short-wavelength sensitive
UVS: ultraviolet sensitive
