# Supplementary material for: A cure for the blues: opsin duplication and subfunctionalization for short-wavelength sensitivity in jewel beetles (Coleoptera: Buprestidae)
Source: BMC Evol Biol. 2016 May 18;16:107. doi: 10.1186/s12862-016-0674-4 (PMC4870758; doi:10.1186/s12862-016-0674-4)
Supplement: Additional file 1: Table S3. — Amino acid sequence comparison of opsin gene copies for selected taxa. (PDF 110 kb) [file 12862_2016_674_MOESM1_ESM.pdf]

Table S3. Amino acid composition and comparison of opsin gene copies (non-ocellar only)

Coleoptera - Selected Comparisons		Sex	Opsin Genes	Aligned Length	Identical amino acids	Conserved amino acids	% similar	Non-conserved amino acids	Gaps
Buprestidae	*Acmaeodera diffusa*	male & female	UVS1 / UVS2	398	227	62	72%	76	33
			LWS1 / LWS2	380	226	62	75%	87	5
	Agrilus planipennis	male & female	UVS1 / UVS2	377	262	47	82%	57	11
			LWS1 / LWS2	380	261	48	81%	68	3
		male & female	LWS1 / LWS3*	326**	208	45	78%	72	1
			LWS2 / LWS3*	326**	244	35	86%	45	2
	Chrysobothris lateralis	male & female	UVS1 / UVS2	375	255	51	82%	65	4
			LWS1 / LWS2	381	261	48	81%	69	3
	Chrysochroa tonkinensis	male	UVS1 / UVS2	373	267	44	83%	59	3
			LWS1 / LWS2	380	262	53	83%	64	1
	Sphenoptera sp.	female	UVS1 / UVS2	377	271	41	83%	58	7
			LWS1 / LWS2	386	269	44	81%	65	8
	Steraspis amplipennis	female	UVS1 / UVS2	258**	180	38	84%	39	1
			LWS1 / LWS2	380	267	52	83%	60	1
	Acmaeodera diffusa & *Agrilus planipennis*	male & female	UVS1 / UVS1	377	277	37	83%	56	7
			UVS2 / UVS2	391	251	47	76%	70	23
			LWS1 / LWS1	380	284	32	83%	60	1
			LWS2 / LWS2	379	284	44	87%	49	2
	Agrilus planipennis & *Chrysochroa tonkinensis*	male & female	UVS1 / UVS1	376	301	33	89%	34	8
			UVS2 / UVS2	372	296	31	88%	43	2
			LWS1 / LWS1	380	288	41	87%	49	2
			LWS2 / LWS2	380	335	27	95%	16	2
	Agrilus planipennis & *Chrysobothris lateralis*	male & female	UVS1 / UVS1	377	303	33	89%	32	9
			UVS2 / UVS2	372	300	33	90%	39	0
			LWS1 / LWS1	381	303	27	87%	47	4
			LWS2 / LWS2	380	332	23	93%	23	2
Dytiscidae	*Thermonectus marmoratus*	larval	UVS1 / UVS2	378	289	36	86%	48	5
Buprestidae / Dytiscidae	*Agrilus planipennis* & *Allodessus bistrigatus*	male / adult	LWS3* / LWS	300**	259	20	93%	21	0
Buprestidae / Dytiscidae	*Agrilus planipennis* & *Limbodessus palmulaoides*	male / adult	LWS3* / LWS	300**	259	20	93%	21	0
Buprestidae / Dytiscidae	*Agrilus planipennis* & *Paroster nigroadumbratus*	male / adult	LWS3* / LWS	300**	257	20	92%	23	0
Buprestidae / Dytiscidae	*Agrilus planipennis* & *Thermonectus marmoratus*	male / adult	LWS3* / LWS	326**	270	20	89%	35	1
Buprestidae / Lampyridae	*Agrilus planipennis* & *Luciola cruciata*	male / adult	LWS3* / LWS	326**	249	31	86%	44	2
Buprestidae / Tenebrionidae	*Agrilus planipennis* & *Tribolium castaneum*	male / adult	LWS3* / LWS	327**	271	23	90%	31	2

* LWS3 in male EAB only

**Normalized for partial sequences. Sequences were aligned and then trimmed for direct comparison.

Insecta - Selected Comparisons of Duplications		Sex	Opsin Genes	Aligned Length	Identical amino acids	Conserved amino acids	% similar	Non-conserved amino acids	Gaps
Diptera	*Drosophila melanogaster*	N/A	UVS Rh3 / UVS Rh4	385	277	35	81%	64	9
Lepidoptera	*Heliconius erato*		UVS1 / UVS2	378	325	29	94%	24	0
	Heliconius melpomene		UVS1 / UVS2	378	334	23	94%	21	0
	Heliconius sapho		UVS1 / UVS2	378	319	31	93%	27	1
	Pieris rapae		blue / violet	380	289	38	86%	48	5
Hemiptera	*Laodelphax striallata*		UVS1 / UVS2	380	318	33	92%	28	1
	Nilaparvata lugens		UVS1 / UVS2	381	312	36	91%	31	2
	Sogatella furcifera		UVS1 / UVS2	386	320	34	92%	25	7
