# Supplementary material for: A cure for the blues: opsin duplication and subfunctionalization for short-wavelength sensitivity in jewel beetles (Coleoptera: Buprestidae)
Source: BMC Evol Biol. 2016 May 18;16:107. doi: 10.1186/s12862-016-0674-4 (PMC4870758; doi:10.1186/s12862-016-0674-4)
Supplement: Additional file 2: Table S4. — Branches and sites under positive selection. P = 95–99. If bolded, P= >99. Amino acid residues are numbered according to bovine rhodopsin. (PDF 176 kb) [file 12862_2016_674_MOESM2_ESM.pdf]

Table S4. Branches and sites under positive selection. P=95-99. If bolded, P= >99.
Amino acid residues are numbered according to bovine rhodopsin.

Coleoptera UVS (branch UV-A). P-value = 1.927345e-08

Group	Organism	Sex	Opsin class	A.A.														
				5*	21**	**9**	48	51	**52**	130	**137**	**151**	169	**186**	199	224	**297**	321‡
Artiodactyla	*Bos taurus* (bovine)	–	rhodopsin	-	-	F	I	G	F	V	V	N	A	S	N	G	T	-
Buprestidae	***Acmaeodera diffusa***	M/F	UVS1	W	H	**S**	F	S	**T**	C	**T**	**I**	I	**T**	D	T	**F**	S
	***Agrilus planipennis* (EAB)**	M/F	UVS1	-	G	**S**	F	S	**T**	C	**T**	**T**	L	**A**	D	S	**C**	A
	Chrysobothris lateralis	M/F	UVS1	W	E	**S**	F	S	**T**	C	**T**	**T**	V	**A**	D	S	**C**	A
	Chrysochroa tonkinensis	M	UVS1	G	E	**S**	F	S	**T**	C	**T**	**T**	V	**A**	D	S	**C**	A
	***Sphenoptera* sp.**	F	UVS1	W	G	**S**	F	S	**T**	C	**T**	**T**	V	**A**	D	S	**C**	A
	Steraspis amplipennis	F	UVS1	T	E	**S**	F	S	**T**	C	**T**	**T**	V	**A**	D	S	**C**	A
	Acmaeodera diffusa	M/F	UVS2	W	S	**E**	F	S	**I**	C	**H**	**T**	I	**Q**	D	I	**T**	G
	***Agrilus planipennis* (EAB)**	M/F	UVS2	W	P	**S**	F	S	**T**	C	**H**	**T**	V	**Q**	D	S	**L**	A
	Chrysobothris lateralis	M/F	UVS2	W	P	**A**	F	S	**I**	C	**H**	**T**	I	**Q**	D	S	**L**	A
	Chrysochroa tonkinensis	M	UVS2	Y	T	**A**	F	S	**T**	C	**H**	**T**	I	**Q**	D	S	**L**	A
	***Sphenoptera* sp.**	F	UVS2	W	P	**A**	F	S	**M**	C	**H**	**T**	V	**Q**	D	S	**M**	A
	Steraspis amplipennis	F	UVS2	Y	P	**S**	F	S	**T**	C	**H**	**T**	V	**Q**	D	S	**M**	A
Coleoptera	***Allodessus bistrigatus***		UVS	-	-	**H**	F	S	**M**	C	**T**	**T**	V	**A**	D	S	**-**	A
	Paroster nigroadumbratus		UVS	-	-	**S**	F	S	**I**	C	**T**	**T**	V	**A**	D	S	**L**	A
	Luciola cruciata		UVS	A	S	**S**	C	G	**I**	F	**A**	**T**	V	**S**	D	S	**F**	A
	Thermonectus marmoratus		UVS1	W	P	**S**	F	S	**L**	C	**T**	**T**	I	**A**	D	S	**L**	A
	Thermonectus marmoratus		UVS2	W	P	**S**	F	S	**M**	C	**N**	**T**	I	**A**	D	S	**M**	A
	Tribolium castaneum		UVS	-	-	**D**	F	A	**T**	C	**T**	**T**	V	**A**	D	S	**F**	A

* Before start of bovine sequence - original residue W5 in *Acmaeodera diffusa* UVS1
** Before start of bovine sequence - original residue H21 in *Acmaeodera diffusa* UVS1
‡ In bovine gap between residues 321 and 322 - original residue S352 in *Acmaeodera diffusa* UVS1

Buprestidae UVS (branch UV-B). P-value = 3.43639.e-03

Group	Organism	Sex	Opsin class	A.A.						
				38	50	81	141	144	301	308
Artiodactyla	*Bos taurus* (bovine)	–	rhodopsin	S	L	V	K	S	Y	M
Buprestidae	***Acmaeodera diffusa***	M/F	UVS1	F	L	V	N	E	V	L
	***Agrilus planipennis* (EAB)**	M/F	UVS1	F	L	F	N	E	F	L
	Chrysobothris lateralis	M/F	UVS1	F	M	F	N	E	F	L
	Chrysochroa tonkinensis	M	UVS1	F	M	F	N	E	L	L
	***Sphenoptera* sp.**	F	UVS1	F	M	F	N	E	F	L
	Steraspis amplipennis	F	UVS1	F	M	F	N	E	-	L
	Acmaeodera diffusa	M/F	UVS2	L	M	F	N	E	L	L
	***Agrilus planipennis* (EAB)**	M/F	UVS2	F	M	F	N	E	L	L
	Chrysobothris lateralis	M/F	UVS2	F	M	F	N	E	L	L
	Chrysochroa tonkinensis	M	UVS2	L	M	F	N	E	L	L
	***Sphenoptera* sp.**	F	UVS2	F	M	I	N	E	L	L
	Steraspis amplipennis	F	UVS2	F	M	F	N	E	L	L
Coleoptera	***Allodessus bistrigatus***		UVS	Y	F	I	K	D	-	-
	Paroster nigroadumbratus		UVS	Y	F	I	K	D	I	I
	Luciola cruciata		UVS	Y	M	F	N	E	L	L
	Thermonectus marmoratus		UVS1	Y	F	F	R	D	I	I
	Thermonectus marmoratus		UVS2	Y	A	I	R	D	I	I
	Tribolium castaneum		UVS	F	M	I	R	D	L	I

Buprestidae UVS1 (branch UV-C). P-value = 1.997754e-03

Group	Organism	Sex	Opsin class	A.A.				
				**21***	105	115	**258**	294
Artiodactyla	*Bos taurus* (bovine)	–	rhodopsin	-	F	F	V	F
Buprestidae	***Acmaeodera diffusa***	M/F	UVS1	**H**	A	T	**T**	C
	***Agrilus planipennis* (EAB)**	M/F	UVS1	**G**	T	T	**T**	F
	Chrysobothris lateralis	M/F	UVS1	**E**	T	T	**T**	F
	Chrysochroa tonkinensis	M	UVS1	**E**	T	M	**T**	F
	***Sphenoptera* sp.**	F	UVS1	**G**	T	T	**T**	F
	Steraspis amplipennis	F	UVS1	**E**	T	A	**T**	F
	Acmaeodera diffusa	M/F	UVS2	S	L	I	C	F
	***Agrilus planipennis* (EAB)**	M/F	UVS2	P	L	V	C	T
	Chrysobothris lateralis	M/F	UVS2	P	T	V	C	T
	Chrysochroa tonkinensis	M	UVS2	T	G	V	C	A
	***Sphenoptera* sp.**	F	UVS2	P	T	V	C	T
	Steraspis amplipennis	F	UVS2	P	T	V	C	T

* Before start of bovine sequence - original residue H21 in *Acmaeodera diffusa* UVS1

Buprestidae UVS2 (branch UV-D). P-value = 6.904998e-04

Group	Organism	Sex	Opsin class	A.A.					
				1*	107	**137**	**186**	207	**242****
Artiodactyla	*Bos taurus* (bovine)	–	rhodopsin	-	P	V	S	M	-
Buprestidae	***Acmaeodera diffusa***	M/F	UVS1	M	H	T	T	I	A
	***Agrilus planipennis* (EAB)**	M/F	UVS1	M	H	T	A	I	A
	Chrysobothris lateralis	M/F	UVS1	M	H	T	A	I	S
	Chrysochroa tonkinensis	M	UVS1	-	H	T	A	I	T
	***Sphenoptera* sp.**	F	UVS1	M	H	T	A	I	T
	Steraspis amplipennis	F	UVS1	-	H	T	A	I	-
	Acmaeodera diffusa	M/F	UVS2	R	M	**H**	**Q**	L	**Q**
	***Agrilus planipennis* (EAB)**	M/F	UVS2	N	Y	**H**	**Q**	L	**Q**
	Chrysobothris lateralis	M/F	UVS2	H	F	**H**	**Q**	L	**Q**
	Chrysochroa tonkinensis	M	UVS2	H	F	**H**	**Q**	L	**Q**
	***Sphenoptera* sp.**	F	UVS2	Y	F	**H**	**Q**	L	**Q**
	Steraspis amplipennis	F	UVS2	H	F	**H**	**Q**	L	**-**

* Before start of bovine sequence - original residue M1 in *Acmaeodera diffusa* UVS1, R18 in *Acmaeodera diffusa* UVS2
** In bovine gap between residues 242 and 243 - original residue A267 in *Acmaeodera diffusa* UVS1

***Acmaeodera diffusa* UVS1 (branch UV-E). P-value = 8.266879e-03**

Group	Organism	Sex	Opsin class	A.A.
				23*
Artiodactyla	*Bos taurus* (bovine)	–	rhodopsin	-
Buprestidae	***Acmaeodera diffusa***	M/F	UVS1	V
	***Agrilus planipennis* (EAB)**	M/F	UVS1	Q
	Chrysobothris lateralis	M/F	UVS1	Q
	Chrysochroa tonkinensis	M	UVS1	R
	***Sphenoptera* sp.**	F	UVS1	Q
	Steraspis amplipennis	F	UVS1	R
	Acmaeodera diffusa	M/F	UVS2	P
	***Agrilus planipennis* (EAB)**	M/F	UVS2	N
	Chrysobothris lateralis	M/F	UVS2	H
	Chrysochroa tonkinensis	M	UVS2	R
	***Sphenoptera* sp.**	F	UVS2	H
	Steraspis amplipennis	F	UVS2	Q

* Before start of bovine sequence - original residue V23 in *Acmaeodera diffusa* UVS1

***Acmaeodera diffusa* UVS2 (branch UV-F). P-value = 4.83125463e-01**																				
**Group**	**Organism**	**Sex**	**Opsin class**	**A.A.**																
				12	**31**	34	82	104	105	111	119	125	161	172	188	196	272	293	294	297
Artiodactyla	*Bos taurus* (bovine)	–	rhodopsin	P	L	P	A	V	F	N	L	L	W	L	G	E	A	F	F	T
Buprestidae	***Acmaeodera diffusa***	M/F	UVS1	V	E	A	L	A	A	Q	L	G	W	F	S	D	S	C	C	F
	***Agrilus planipennis* (EAB)**	M/F	UVS1	S	E	P	C	A	T	Q	L	G	W	L	S	D	S	C	F	C
	Chrysobothris lateralis	M/F	UVS1	V	E	A	C	A	T	Q	L	G	W	L	S	D	A	C	F	C
	Chrysochroa tonkinensis	M	UVS1	V	E	A	C	A	T	Q	L	G	W	L	S	D	A	C	F	C
	***Sphenoptera* sp.**	F	UVS1	V	E	A	C	A	T	Q	L	G	W	L	T	D	A	C	F	C
	Steraspis amplipennis	F	UVS1	V	E	A	C	A	T	Q	I	G	W	G	S	D	-	-	-	-
	Acmaeodera diffusa	M/F	UVS2	P	**P**	M	L	Y	L	Y	Y	S	Y	Y	G	L	C	L	F	T
	***Agrilus planipennis* (EAB)**	M/F	UVS2	G	E	A	C	T	L	Q	L	G	W	L	T	R	A	C	T	L
	Chrysobothris lateralis	M/F	UVS2	V	E	A	C	A	T	Q	L	S	W	L	T	K	A	C	T	L
	Chrysochroa tonkinensis	M	UVS2	E	A	A	S	A	G	Q	L	S	W	L	T	N	A	C	A	L
	***Sphenoptera* sp.**	F	UVS2	V	E	A	C	A	T	Q	L	G	W	L	S	T	A	C	T	M
	Steraspis amplipennis	F	UVS2	V	E	A	C	A	T	Q	L	G	W	L	S	N	A	C	T	M

**Coleoptera (majority) LWS (branch LW-A). P-value = 1.3228e-05**					
**Group**	**Organism**	**Sex**	**Opsin class**	**A.A.**	
				**144**	**315**
Artiodactyla	*Bos taurus* (bovine)	–	rhodopsin	S	N
Buprestidae	***Acmaeodera diffusa***	M/F	LWS1	A	Q
	***Agrilus planipennis* (EAB)**	M/F	LWS1	S	Q
	Chrysobothris lateralis	M/F	LWS1	S	Q
	Chrysochroa tonkinensis	M	LWS1	S	Q
	***Sphenoptera* sp.**	F	LWS1	S	Q
	Steraspis amplipennis	F	LWS1	S	Q
	Acmaeodera diffusa	M/F	LWS2	S	Q
	***Agrilus planipennis* (EAB)**	M/F	LWS2	S	Q
	Chrysobothris lateralis	M/F	LWS2	S	Q
	Chrysochroa tonkinensis	M	LWS2	S	Q
	***Sphenoptera* sp.**	F	LWS2	S	Q
	Steraspis amplipennis	F	LWS2	S	Q
	***Agrilus planipennis* (EAB)**	M	LWS3	S	A
Coleoptera	***Allodessus bistrigatus***		LWS	S	A
	Limbodessus palmulaoides		LWS	S	A
	Paroster nigroadumbratus		LWS	S	A
	Luciola cruciata		LWS	S	Q
	Thermonectus marmoratus		LWS	S	A
	Tribolium castaneum		LWS	S	Q

**Buprestidae LWS (branch LW-B). P-value = 1.633356e-06**								
**Group**	**Organism**	**Sex**	**Opsin class**	**A.A.**				
				16*	63	242**	**273**	**280**
Artiodactyla	*Bos taurus* (bovine)	–	rhodopsin	-	V	-	F	G
Buprestidae	***Acmaeodera diffusa***	M/F	LWS1	R	S	G	**L**	**L**
	***Agrilus planipennis* (EAB)**	M/F	LWS1	R	S	G	**V**	**L**
	Chrysobothris lateralis	M/F	LWS1	R	S	G	**V**	**L**
	Chrysochroa tonkinensis	M	LWS1	R	S	G	**L**	**L**
	***Sphenoptera* sp.**	F	LWS1	R	S	G	**C**	**L**
	Steraspis amplipennis	F	LWS1	R	S	G	**L**	**V**
	Acmaeodera diffusa	M/F	LWS2	R	C	G	**W**	**P**
	***Agrilus planipennis* (EAB)**	M/F	LWS2	R	S	G	**W**	**T**
	Chrysobothris lateralis	M/F	LWS2	R	T	G	**W**	**T**
	Chrysochroa tonkinensis	M	LWS2	R	S	G	**Y**	**T**
	***Sphenoptera* sp.**	F	LWS2	R	T	G	**W**	**T**
	Steraspis amplipennis	F	LWS2	R	S	G	**Y**	**T**
	***Agrilus planipennis* (EAB)**	M	LWS3	-	S	S	Y	G
Coleoptera	***Allodessus bistrigatus***		LWS	-	S	G	F	G
	Limbodessus palmulaoides		LWS	-	S	G	F	G
	Paroster nigroadumbratus		LWS	-	S	S	W	G
	Luciola cruciata		LWS	A	S	S	Y	G
	Thermonectus marmoratus		LWS	-	S	G	Y	G
	Tribolium castaneum		LWS	-	S	S	F	G

* Before start of bovine sequence - original residue R16 in *Acmaeodera diffusa* LWS1
** In bovine gap between residues 242 and 243 - original residue G266 in *Acmaeodera diffusa* LWS1

**Buprestidae LWS1 + *Acmaeodera diffusa* LWS2 (branch LW-C). P-value = 4.604520e-02**						
**Group**	**Organism**	**Sex**	**Opsin class**	**A.A.**		
				51	263	274
Artiodactyla	*Bos taurus* (bovine)	–	rhodopsin	G	I	Y
Buprestidae	***Acmaeodera diffusa***	M/F	LWS1	S	I	T
	***Agrilus planipennis* (EAB)**	M/F	LWS1	A	M	V
	Chrysobothris lateralis	M/F	LWS1	C	I	V
	Chrysochroa tonkinensis	M	LWS1	A	M	A
	***Sphenoptera* sp.**	F	LWS1	A	M	V
	Steraspis amplipennis	F	LWS1	A	M	V
	Acmaeodera diffusa	M/F	LWS2	A	I	L
	***Agrilus planipennis* (EAB)**	M/F	LWS2	S	F	T
	Chrysobothris lateralis	M/F	LWS2	S	F	T
	Chrysochroa tonkinensis	M	LWS2	S	F	T
	***Sphenoptera* sp.**	F	LWS2	S	F	T
	Steraspis amplipennis	F	LWS2	S	F	T
	***Agrilus planipennis* (EAB)**	M	LWS3	S	F	T

**Buprestidae LWS1 (branch LW-E). P-value = 5.534318e-02**										
**Group**	**Organism**	**Sex**	**Opsin class**	**A.A.**						
				46	**111**	**123**	**211**	**242***	273	321**
Artiodactyla	*Bos taurus* (bovine)	–	rhodopsin	L	N	I	H	-	F	-
Buprestidae	***Acmaeodera diffusa***	M/F	LWS1	T	**Q**	**V**	**C**	**A**	L	S
	***Agrilus planipennis* (EAB)**	M/F	LWS1	C	**N**	**V**	**C**	**Q**	V	K
	Chrysobothris lateralis	M/F	LWS1	T	**N**	**V**	**C**	**Q**	V	S
	Chrysochroa tonkinensis	M	LWS1	T	**T**	**A**	**C**	**Q**	L	K
	***Sphenoptera* sp.**	F	LWS1	T	**N**	**V**	**C**	**Q**	C	S
	Steraspis amplipennis	F	LWS1	I	**Q**	**V**	**C**	**Q**	L	K
	Acmaeodera diffusa	M/F	LWS2	V	E	T	V	A	W	A
	***Agrilus planipennis* (EAB)**	M/F	LWS2	I	E	T	V	A	W	S
	Chrysobothris lateralis	M/F	LWS2	V	Q	T	C	A	W	S
	Chrysochroa tonkinensis	M	LWS2	V	E	T	V	A	Y	A
	***Sphenoptera* sp.**	F	LWS2	V	E	T	V	A	W	S
	Steraspis amplipennis	F	LWS2	V	E	T	V	A	Y	A
	***Agrilus planipennis* (EAB)**	M	LWS3	V	E	I	V	A	Y	A

* In bovine gap between residues 242 and 243 - original residue A268 in *Acmaeodera diffusa* LWS1
** In bovine gap between residues 321 and 322 - original residue S351 in *Acmaeodera diffusa* LWS1

Acmaeodera diffusa LWS2 (branch LW-F). P-value = 9.972361e-01					
**Group**	**Organism**	**Sex**	**Opsin class**	**A.A.**	
				93	297
Artiodactyla	*Bos taurus* (bovine)	–	rhodopsin	T	T
	Acmaeodera diffusa	M/F	LWS1	P	A
	***Agrilus planipennis* (EAB)**	M/F	LWS1	P	A
	Chrysobothris lateralis	M/F	LWS1	P	A
	Chrysochroa tonkinensis	M	LWS1	P	A
	***Sphenoptera* sp.**	F	LWS1	P	A
	Steraspis amplipennis	F	LWS1	P	A
Buprestidae	***Acmaeodera diffusa***	M/F	LWS2	E	M
	***Agrilus planipennis* (EAB)**	M/F	LWS2	P	A
	Chrysobothris lateralis	M/F	LWS2	P	A
	Chrysochroa tonkinensis	M	LWS2	P	A
	***Sphenoptera* sp.**	F	LWS2	P	A
	Steraspis amplipennis	F	LWS2	P	A
	***Agrilus planipennis* (EAB)**	M	LWS3	A	A

Acmaeodera diffusa LWS1 (branch LW-G). P-value = 2.900492e-03							
**Group**	**Organism**	**Sex**	**Opsin class**	**A.A.**			
				144	156	159	242*
Artiodactyla	*Bos taurus* (bovine)	–	rhodopsin	S	G	F	-
	Acmaeodera diffusa	M/F	LWS1	A	W	N	A
	***Agrilus planipennis* (EAB)**	M/F	LWS1	S	W	M	Q
	Chrysobothris lateralis	M/F	LWS1	S	Q	L	Q
	Chrysochroa tonkinensis	M	LWS1	S	Q	L	Q
	***Sphenoptera* sp.**	F	LWS1	S	Q	W	Q
	Steraspis amplipennis	F	LWS1	S	Q	L	Q
Buprestidae	***Acmaeodera diffusa***	M/F	LWS2	S	R	I	A
	***Agrilus planipennis* (EAB)**	M/F	LWS2	S	R	L	A
	Chrysobothris lateralis	M/F	LWS2	S	R	I	A
	Chrysochroa tonkinensis	M	LWS2	S	R	L	A
	***Sphenoptera* sp.**	F	LWS2	S	R	L	A
	Steraspis amplipennis	F	LWS2	S	R	L	A
	***Agrilus planipennis* (EAB)**	M	LWS3	S	Q	A	A

* In bovine gap between residues 242 and 243 - original residue A268 in *Acmaeodera diffusa* LWS1

**Coleoptera LWS (some) +*Agrilus planipennis* LWS3 (male) (branch LW-H). P-value = 5.296171e-07**												
**Group**	**Organism**	**Sex**	**Opsin class**	**A.A.**								
				**123**	**156**	**170**	197	**243**	253	**281**	316	**318**
Artiodactyla	*Bos taurus* (bovine)	–	rhodopsin	I	G	P	E	T	M	S	C	V
	Acmaeodera diffusa	M/F	LWS1	V	W	A	D	N	V	A	A	Y
	***Agrilus planipennis* (EAB)**	M/F	LWS1	V	W	A	D	N	V	A	A	Y
	Chrysobothris lateralis	M/F	LWS1	V	Q	A	D	N	V	F	A	Y
	Chrysochroa tonkinensis	M	LWS1	A	Q	A	N	N	V	A	A	Y
	***Sphenoptera* sp.**	F	LWS1	V	Q	A	D	N	V	A	A	Y
	Steraspis amplipennis	F	LWS1	V	Q	A	D	N	V	A	A	Y
Buprestidae	***Acmaeodera diffusa***	M/F	LWS2	T	R	A	D	K	V	S	A	Y
	***Agrilus planipennis* (EAB)**	M/F	LWS2	T	R	A	D	N	V	A	A	H
	Chrysobothris lateralis	M/F	LWS2	T	R	A	D	N	V	A	A	H
	Chrysochroa tonkinensis	M	LWS2	T	R	A	D	N	V	A	A	Y
	***Sphenoptera* sp.**	F	LWS2	T	R	A	N	N	V	A	A	Y
	Steraspis amplipennis	F	LWS2	T	R	A	D	N	V	A	A	Y
	***Agrilus planipennis* (EAB)**	M	LWS3	**I**	**Q**	**L**	E	**Q**	I	**K**	V	**L**
	Allodessus bistrigatus		LWS	**I**	**Q**	**F**	E	**A**	I	**K**	V	**M**
	Limbodessus palmulaoides		LWS	**I**	**Q**	**F**	E	**A**	I	**K**	V	**M**
	Paroster nigroadumbratus		LWS	**I**	**Q**	**F**	D	**Q**	I	**K**	V	**M**
Coleoptera	***Luciola cruciata***		LWS	**T**	**R**	**A**	G	**Q**	V	**S**	A	**Q**
	Thermonectus marmoratus		LWS	**I**	**Q**	**F**	E	**Q**	I	**K**	E	**F**
	Tribolium castaneum		LWS	**A**	**R**	**A**	D	**Q**	I	**A**	A	**Q**
