# Supplementary material for: A cure for the blues: opsin duplication and subfunctionalization for short-wavelength sensitivity in jewel beetles (Coleoptera: Buprestidae)
Source: BMC Evol Biol. 2016 May 18;16:107. doi: 10.1186/s12862-016-0674-4 (PMC4870758; doi:10.1186/s12862-016-0674-4)
Supplement: Additional file 3: Table S5. — Log-likelihood values and parameter estimates of branch-site tests of positive selection. (PDF 117 kb) [file 12862_2016_674_MOESM3_ESM.pdf]

Table S5. Log-likelihood values and parameter estimates of branch-site tests of positive selection.

Branch	P-value	Model	λ	Site class	Proportion	Background ω	Foreground ω
**Coleoptera UVS (UV-A)**	**1.93E-08**	**A**	-89957.54	0	0.82442	0.0808	0.0808
				1	0.09211	1	1
				2a	0.07508	0.0808	999
				2b	0.00839	1	999
		**Null**	-89973.32	0	0.68836	0.08071	0.08071
				1	0.07689	1	1
				2a	0.21116	0.08071	1
				2b	0.02359	1	1

Branch	P-value	Model	λ	Site class	Proportion	Background ω	Foreground ω
**Buprestidae UVS (UV-B)**	**3.44E-03**	**A**	-89978.32	0	0.82406	0.08084	0.08084
				1	0.09209	1	1
				2a	0.07542	0.08084	999
				2b	0.00843	1	999
		**Null**	-89982.6	0	0	0.08082	0.08082
				1	0	1	1
				2a	0.89971	0.08082	1
				2b	0.10029	1	1

Branch	P-value	Model	λ	Site class	Proportion	Background ω	Foreground ω
**Buprestidae UVS1 (UV-C)**	**2.00E-03**	**A**	-89976.76	0	0.85273	0.0809	0.0809
				1	0.09587	1	1
				2a	0.0462	0.0809	39.61114
				2b	0.00519	1	39.61114
		**Null**	-89981.54	0	0.80478	0.08074	0.08074
				1	0.09029	1	1
				2a	0.09434	0.08074	1
				2b	0.01058	1	1

Branch	P-value	Model	λ	Site class	Proportion	Background ω	Foreground ω
**Buprestidae UVS2 (UV-D)**	**6.90E-04**	**A**	-89976.01	0	0.83912	0.08069	0.08069
				1	0.09409	1	1
				2a	0.06006	0.08069	22.38806
				2b	0.00673	1	22.38806
		**Null**	-89981.77	0	0.76673	0.08078	0.08078
				1	0.08581	1	1
				2a	0.13262	0.08078	1
				2b	0.01484	1	1

Branch	P-value	Model	λ	Site class	Proportion	Background ω	Foreground ω
***Acmaeodera diffusa* UVS1 (UV-E)**	**8.27E-03**	**A**	-89984.9	0	0.8711	0.08093	0.08093
				1	0.09741	1	1
				2a	0.02832	0.08093	5.411
				2b	0.00317	1	5.411
		**Null**	-89988.39	0	0.89947	0.0811	0.0811
				1	0.10053	1	1
				2a	0	0.0811	1
				2b	0	1	1

Branch	P-value	Model	λ	Site class	Proportion	Background ω	Foreground ω
Acmaeodera diffusa UVS2 (UV-F)	4.83E-01	A	-89967.07	0	0.79313	0.08058	0.08058
				1	0.08887	1	1
				2a	0.10611	0.08058	18.0692
				2b	0.01189	1	18.0692
		Null	-89967.27	0	0.73936	0.08032	0.08032
				1	0.08268	1	1
				2a	0.16006	0.08032	1
				2b	0.0179	1	1

Branch	P-value	Model	λ	Site class	Proportion	Background ω	Foreground ω
**Coleoptera (majority) LWS (LW-A)**	1.32E-05	A	-89974.65	0	0.86677	0.08096	0.08096
				1	0.09584	1	1
				2a	0.03366	0.08096	999
				2b	0.00372	1	999
		Null	-89984.13	0	0.7795	0.08091	0.08091
				1	0.08695	1	1
				2a	0.12015	0.08091	1
				2b	0.0134	1	1

Branch	P-value	Model	λ	Site class	Proportion	Background ω	Foreground ω
**Buprestidae LWS (LW-B)**	1.63E-06	A	-89976.89	0	0.85621	0.08078	0.08078
				1	0.09588	1	1
				2a	0.04308	0.08078	23.57758
				2b	0.00482	1	23.57758
		Null	-89988.39	0	0.89952	0.0811	0.0811
				1	0.10048	1	1
				2a	0	0.0811	1
				2b	0	1	1

Branch	P-value	Model	λ	Site class	Proportion	Background ω	Foreground ω
**Buprestidae LWS1 + *Acmaeodera diffusa* LWS2 (LW-C)**	4.60E-02	A	-89986.4	0	0.85191	0.08088	0.08088
				1	0.09524	1	1
				2a	0.04754	0.08088	88.91785
				2b	0.00531	1	88.91785
		Null	-89988.39	0	0.89952	0.0811	0.0811
				1	0.10048	1	1
				2a	0	0.0811	1
				2b	0	1	1

Branch	P-value	Model	λ	Site class	Proportion	Background ω	Foreground ω
**Buprestidae LWS2 - *Acmaeodera diffusa* LWS2 (LW-D)**	6.23E-01	A	-89987.21	0	0.86811	0.08089	0.08089
				1	0.09692	1	1
				2a	0.03146	0.08089	5.45675
				2b	0.00351	1	5.45675
		Null	-89987.33	0	0.74585	0.08087	0.08087
				1	0.08328	1	1
				2a	0.15371	0.08087	1
				2b	0.01716	1	1

Branch	P-value	Model	λ	Site class	Proportion	Background ω	Foreground ω
**Buprestidae LWS1 (LW-E)**	**5.53E-02**	**A**	-89975.11	0	0.71767	0.08058	0.08058
				1	0.08005	1	1
				2a	0.18198	0.08058	4.36911
				2b	0.0203	1	4.36911
		**Null**	-89976.94	0	0.66043	0.08062	0.08062
				1	0.0738	1	1
				2a	0.23906	0.08062	1
				2b	0.02671	1	1

Branch	P-value	Model	λ	Site class	Proportion	Background ω	Foreground ω
Acmaeodera diffusa LWS2 (LW-F)	**9.97E-01**	**A**	-89984.41	0	0.82406	0.08079	0.08079
				1	0.09222	1	1
				2a	0.07529	0.08079	1
				2b	0.00843	1	1
		**Null**	-89984.41	0	0.82405	0.08079	0.08079
				1	0.09225	1	1
				2a	0.07527	0.08079	1
				2b	0.00843	1	1

Branch	P-value	Model	λ	Site class	Proportion	Background ω	Foreground ω
Acmaeodera diffusa LWS1 (LW-G)	**2.90E-03**	**A**	-89975.36	0	0.85633	0.08076	0.08076
				1	0.09579	1	1
				2a	0.04306	0.08076	11.95684
				2b	0.00482	1	11.95684
		**Null**	-89979.8	0	0.84052	0.08065	0.08065
				1	0.09398	1	1
				2a	0.05891	0.08065	1
				2b	0.00659	1	1

Branch	P-value	Model	λ	Site class	Proportion	Background ω	Foreground ω
**Coleoptera LWS +** *Agrilus planipennis* **LWS3 (male) (LW-H)**	**5.30E-07**	**A**	-89966.89	0	0.84945	0.08062	0.08062
				1	0.09514	1	1
				2a	0.04983	0.08062	999
				2b	0.00558	1	999
		**Null**	-89979.46	0	0.70299	0.0807	0.0807
				1	0.07857	1	1
				2a	0.19648	0.0807	1
				2b	0.02196	1	1
