# Supplementary material for: A cure for the blues: opsin duplication and subfunctionalization for short-wavelength sensitivity in jewel beetles (Coleoptera: Buprestidae)
Source: BMC Evol Biol. 2016 May 18;16:107. doi: 10.1186/s12862-016-0674-4 (PMC4870758; doi:10.1186/s12862-016-0674-4)
Supplement: Additional file 4: Table S1. — Taxon sampling data and GenBank accession numbers (http://www.ncbi.nlm.nih.gov) for gene sequences analyzed in this study. (PDF 128 kb) [file 12862_2016_674_MOESM4_ESM.pdf]

Table S1. Taxon sampling data and GenBank accession numbers (http://www.ncbi.nlm.nih.gov) for gene sequences analyz ed in this study

Class	Order	Family	Binomen	Description	Sequence Length, Amino Acids	Data Source	GenBank Accession #	Comments
Mammalia	Artiodactyla	Bovidae	*Bos taurus*	Bovine rhodopsin	348	GenBank	P02699	outgroup
Cephalopoda	Octapoda	Octapodidae	*Enteroctopus dofleini*	rhodopsin	455	GenBank	X07797	outgroup
	Sepiida	Sepiidae	*Sepia officinalis*	rhodopsin	464		AF000947	outgroup
	Teuthida	Lologinidae	*Doryteuthis pealei*	rhodopsin	442		AY450853	outgroup
		Lologinidae	*Loligo forbesii*	rhodopsin	452		X56788	outgroup
		Ommastrephidae	*Todarodes pacificus*	rhodopsin	448		X70498	outgroup
Insecta	Coleoptera	Buprestidae	*Acmaeodera diffusa* male	LWS opsin 1	378	this paper	KX118640	c6662_g1_i2
				LWS opsin 2	377		KX118641	c36030_g1_i1
				UVS opsin 1	376		KX118642	c40858_g1_i1
				UVS opsin 2	387		KX118643	c33890_g1_i1
			Acmaeodera diffusa female	LWS opsin 1	378	this paper	KX118636	c79530_g1_i1
				LWS opsin 2	377		KX118637	c57341_g1_i1
				UVS opsin 1	376		KX118638	c2583_g1_i1
				UVS opsin 2	387		KX118639	c52170_g1_i1
			Agrilus planipennis male	LWS opsin 1	378	this paper	KX118648	comp9008_C0_seq1
				LWS opsin 2	379		KX118649	comp1871_C0_seq1
				LWS opsin 3, partial CDS	326		KX118650	comp622_C0_seq1
				UVS opsin 1	371		KX118651	comp9013_C0_seq1
				UVS opsin 2	372		KX118652	comp1895_C0_seq1
			Agrilus planipennis female	LWS opsin 1	378	this paper	KX118644	comp7933_C0_seq1
				LWS opsin 2	379		KX118645	comp2366_C0_seq1
				UVS opsin 1	371		KX118646	comp7952_C0_seq1
				UVS opsin 2	372		KX118647	comp2434_C0_seq1
			Chrysobothris lateralis male	LWS opsin 1	380	this paper	KX118657	c13739_g1_i1
				LWS opsin 2	379		KX118658	c25744_g1_i1
				UVS opsin 1	374		KX118659	c26634_g1_i2
				UVS opsin 2	372		KX118660	c24022_g1_i2
			Chrysobothris lateralis female	LWS opsin 1	380	this paper	KX118653	c15801_g1_i1
				LWS opsin 2	379		KX118654	c15923_g1_i1
				UVS opsin 1, partial CDS	354		KX118655	c9244_g1_i2
				UVS opsin 2, partial CDS	365		KX118656	c12991_g1_i1
			Chrysochroa tonkinensis male	LWS opsin 1	380	this paper	KX118661	comp2238_C0_seq1
				LWS opsin 2	379		KX118662	comp6813_C0_seq1
				UVS opsin 1	373		KX118663	comp2873_C0_seq1
				UVS opsin 2	370		KX118664	comp6707_C0_seq1
			Sphenoptera sp. female	LWS opsin 1	385	this paper	KX118665	c15549_g1_i1
				LWS opsin 2	379		KX118666	c41499_g1_i1
				UVS opsin 1	375		KX118667	c9487_g1_i1
				UVS opsin 2	372		KX118668	c26154_g1_i1
			Steraspis amplipennis female	LWS opsin 1	380	this paper	KX118669	c7500_g1_i1
				LWS opsin 2	379		KX118670	c28513_g1_i1
				UVS opsin 1, partial CDS	257		KX118671	c8354_g1_i1
				UVS opsin 2	370		KX118672	c41530_g1_i1
		Tenebrionidae	*Tribolium castaneum*	UVS opsin	358	GenBank	XM_965251	
				LWS opsin	378		NM_001162519	
		Lampyridae	*Luciola cruciata*	LWS opsin	380	GenBank	AB300328	
				UVS opsin	382		AB300329	
		Dytiscidae	*Allodessus bistrigatus*	LWS opsin, partial CDS	328	GenBank	KP219381	
				UVS opsin, partial CDS	249		KP219384	
			Limbodessus palmulaoides	LWS opsin, partial CDS	328	GenBank	KP219382	
			Paroster nigroadumbratus	LWS opsin, partial CDS	328	GenBank	KP219380	
				UVS opsin, partial CDS	321		KP219383	
			Thermonectus marmoratus	LWS opsin	376	GenBank	EU921225	larval specimen
				UVS opsin 1	376		EU921226	larval specimen, instar 1 only
				UVS opsin 2	375		EU921227	larval specimen
	Hymenoptera	Apidae	*Apis cerana*	SWS opsin	377	GenBank	AB355817	
				LWS opsin	377		AB355818	
				UVS opsin	371		AB355816	
			Apis mellifera	SWS opsin	377	GenBank	NM_001011606	
				LWS opsin 1	377		NM_001011639	
				LWS opsin 2	385		NM_001077825	ocellar-specific opsin
				UVS opsin	371		NM_001011605	
		Agaonidae	*Ceratosolen solmsi*	SWS opsin	391	GenBank	JX402132	
				LWS opsin 1	388		JX402130	
				LWS opsin 2	398		JX402131	ocellar-specific opsin
				UVS opsin	374		JX402133	
	Lepidoptera	Nymphalidae	*Heliconius erato*	SWS opsin	380	GenBank	AY918906	
				LWS opsin	380		AY918907	
				UVS opsin 1	378		AY918904	
				UVS opsin 2	378		AY918905	
			Heliconius melpomene	SWS opsin	380	GenBank	GU324689	
				LWS opsin	380		GU324702	
				UVS opsin 1	378		GU324678	
				UVS opsin 2	378		GU324679	
			Heliconius sapho	SWS opsin	380	GenBank	GU324692	
				LWS opsin	380		GU324705	
				UVS opsin 1	377		GQ451907	
				UVS opsin 2	378		GQ451908	
		Sphingidae	*Macroglossum stellatarum*	SWS opsin	383	GenBank	KF539426	
				LWS opsin	381		KF539444	
				UVS opsin	376		KF539456	
			Manduca sexta	SWS opsin	384	GenBank	AD001674	
				LWS opsin	377		L78080	
				UVS opsin	377		L78081	
		Nymphalidae	*Danaus plexippus*	SWS opsin	379	GenBank	AY605544	
				LWS opsin	381		AY605545	
				UVS opsin	377		AY605546	
		Pieridae	*Pieris rapae*	SWS opsin	380	GenBank	AB208675	
				LWS opsin 1	382		AB177984	
				UVS opsin	374		AB208673	
				Violet-sensitive opsin	375		AB208674	
	Odonata	Lestidae	*Indolestes peregrinus*	LWS opsin RhLWA1	378	GenBank	LC009292	
				LWS opsin RhLWA2	380		LC009293	
				LWS opsin RhLWC1	378		LC009294	
				LWS opsin RhLWD1	376		LC009295	
				LWS opsin RhLWD2	380		LC009296	
				LWS opsin RhLWE1	376		LC009297	
				LWS opsin RhLWE2	371		LC009298	
				LWS opsin RhLWF1	371		LC009299	
				SWS opsin RhSWA1	379		LC009300	
				SWS opsin RhSWB1	383		LC009301	
				UVS opsin RhUV	385		LC009302	
		Libelullidae	*Sympetrum frequens*	LWS opsin RhLWA1	378	Genbank	LC009060	
				LWS opsin RhLWA2	380		LC009061	
				LWS opsin RhLWB1	377		LC009062	
				LWS opsin RhLWC1	376		LC009063	
				LWS opsin RhLWD1	366		LC009064	
				LWS opsin RhLWE1	370		LC009065	
				LWS opsin RhLWF1	365		LC009066	
				LWS opsin RhLWF2	372		LC009067	
				LWS opsin RhLWF3	378		LC009068	
				LWS opsin RhLWF4	373		LC009069	
				SWS opsin RhSWA1	388		LC009070	
				SWS opsin RhSWB1	378		LC009071	
				SWS opsin RhSWC1	383		LC009072	
				SWS opsin RhSWC2	382		LC009073	
				SWS opsin RhSWC3	383		LC009074	
				UVS opsin RhUV	390		LC009075	
	Orthoptera	Gryllidae	*Dianemobius nigrofasciatus*	SWS opsin	379	GenBank	AB291232	
				LWS opsin	376		FJ232921	
				UVS opsin	376		AB458852	
			Gryllus bimaculatus	SWS opsin	379	GenBank	HM363622	
				Green-sensitive opsin 1	379		HM363620	ocellar-specific opsin
				Green-sensitive opsin 2	377		HM363621	
				UVS opsin	377		HM363623	
	Hemiptera	Delphacidae	*Laodelphax striatella*	LWS opsin	388	GenBank	AB761153	
				UVS opsin 1	380		AB761154	
				UVS opsin 2	379		AB761155	
		Delphacidae	*Nilaparvata lugens*	LWS opsin	386	GenBank	AB761147	
				UVS opsin 1	380		AB761148	
				UVS opsin 2	380		AB761149	
		Delphacidae	*Sogatella furcifera*	LWS opsin	388	GenBank	AB761150	
				UVS opsin 1	386		AB761151	
				UVS opsin 2	379		AB761152	
		Cicadellidae	*Nephotettix cincticeps*	SWS opsin	377	GenBank	AB761157	
				LWS opsin	379		AB761156	
				UVS opsin	380		AB761158	
		Aphididae	*Megoura viciae*	LWS opsin	378	GenBank	AF189714	
				UVS opsin	371		AF189715	
	Diptera	Drosophilidae	*Drosophila melanogaster*	SWS opsin Rh5	382	FlyBase	FlyBase Annotation ID: CG5279	
				LWS opsin Rh2	381		FlyBase Annotation ID: CG16740	ocellar-specific opsin
				LWS opsin Rh1	373		FlyBase Annotation ID: CG4550	
				UVS opsin Rh3	383		FlyBase Annotation ID: CG10888	
				UVS opsin Rh4	378		FlyBase Annotation ID: CG9668	
				LWS opsin Rh6	369	GenBank	Z86118	
