# Supplementary material for: A cure for the blues: opsin duplication and subfunctionalization for short-wavelength sensitivity in jewel beetles (Coleoptera: Buprestidae)
Source: BMC Evol Biol. 2016 May 18;16:107. doi: 10.1186/s12862-016-0674-4 (PMC4870758; doi:10.1186/s12862-016-0674-4)
Supplement: Additional file 5: Table S2. — Statistics for transcriptomes generated in this study. (PDF 91 kb) [file 12862_2016_674_MOESM5_ESM.pdf]

Table S2. Statistics for transcriptomes generated in this study

Order	Subfamily	Taxon Name	Sex	Longest Contig	Number of Contigs	N50 Value
Coleoptera	Polycestinae	*Acmaeodera diffusa*	male	27521	142298	414
Coleoptera	Polycestinae	*Acmaeodera diffusa*	female	21503	197926	427
Coleoptera	Agrilinae	*Agrilus planipennis*	male	17967	52315	3276
Coleoptera	Agrilinae	*Agrilus planipennis*	female	19263	44591	2931
Coleoptera	Buprestinae	*Chrysobothris lateralis*	male	17894	97466	513
Coleoptera	Buprestinae	*Chrysobothris lateralis*	female	21488	77748	583
Coleoptera	Chrysochroinae	*Chrysochroa tonkinensis*	male	25313	63672	1789
Coleoptera	Chrysochroinae	*Sphenoptera* sp.	female	16306	68535	478
Coleoptera	Chrysochroinae	*Steraspis amplipennis*	female	17176	49956	661
